# Bidirectional Regulation of Innate and Learned Behaviors That Rely on Frequency Discrimination by Cortical Inhibitory Neurons

**DOI:** 10.1371/journal.pbio.1002308

**Published:** 2015-12-02

**Authors:** Mark Aizenberg, Laetitia Mwilambwe-Tshilobo, John J. Briguglio, Ryan G. Natan, Maria N. Geffen

**Affiliations:** 1 Department of Otorhinolaryngology HNS, Neuroscience Graduate Group, University of Pennsylvania, Philadelphia, Pennsylvania, United States of America; 2 Physics Graduate Group, University of Pennsylvania, Philadelphia, Pennsylvania, United States of America; New York University School of Medicine, UNITED STATES

## Abstract

The ability to discriminate tones of different frequencies is fundamentally important for everyday hearing. While neurons in the primary auditory cortex (AC) respond differentially to tones of different frequencies, whether and how AC regulates auditory behaviors that rely on frequency discrimination remains poorly understood. Here, we find that the level of activity of inhibitory neurons in AC controls frequency specificity in innate and learned auditory behaviors that rely on frequency discrimination. Photoactivation of parvalbumin-positive interneurons (PVs) improved the ability of the mouse to detect a shift in tone frequency, whereas photosuppression of PVs impaired the performance. Furthermore, photosuppression of PVs during discriminative auditory fear conditioning increased generalization of conditioned response across tone frequencies, whereas PV photoactivation preserved normal specificity of learning. The observed changes in behavioral performance were correlated with bidirectional changes in the magnitude of tone-evoked responses, consistent with predictions of a model of a coupled excitatory-inhibitory cortical network. Direct photoactivation of excitatory neurons, which did not change tone-evoked response magnitude, did not affect behavioral performance in either task. Our results identify a new function for inhibition in the auditory cortex, demonstrating that it can improve or impair acuity of innate and learned auditory behaviors that rely on frequency discrimination.

## Author Summary

Hearing perception relies on our ability to tell apart the spectral content of different sounds, and to learn to use this difference to distinguish behaviorally relevant (such as dangerous and safe) sounds. Recently, we demonstrated that the auditory cortex regulates frequency discrimination acuity following associative learning. However, the neuronal circuits that underlie this modulation remain unknown. In the auditory cortex, excitatory neurons serve the dominant function in transmitting information about the sensory world within and across brain areas, whereas inhibitory interneurons carry a range of modulatory functions, shaping the way information is represented and processed. Our study elucidates the function of a specific inhibitory neuronal population in sound encoding and perception. We find that interneurons in the auditory cortex, belonging to a specific class (parvalbumin-positive), modulate frequency selectivity of excitatory neurons, and regulate frequency discrimination acuity and specificity of discriminative auditory associative learning. These results expand our understanding of how specific cortical circuits contribute to innate and learned auditory behavior.

## Abstract 3

Modulating the activity of a specific type of cortical neuron can either improve or impair the ability to discriminate between tones of different frequencies and to associate danger with specific sounds.

## Introduction

Frequency discrimination is a fundamental task in everyday hearing and can be vitally important, as spectral differences can be used to distinguish dangerous and safe sounds [1–3]. However, our knowledge of the neuronal mechanisms that support frequency discrimination remains incomplete. The auditory cortex (AC) is involved in many auditory behaviors [4–13], with some studies suggesting that it controls frequency discrimination [14–16] (but see [5,17]). It remains poorly understood which aspects of neuronal circuits in AC contribute to behavioral frequency discrimination performance.

Neurons in AC exhibit frequency selectivity in their responses to tones [18–24], and modify their tuning properties with auditory learning [25–27], providing support for the involvement of AC in frequency discrimination. Many aspects of neuronal responses to tones, including magnitude of neuronal responses and width of tuning, can in principle affect behavioral performance [28]. Furthermore, learning and experience can lead to changes in tone-evoked response patterns in AC [25,27,29–31], affecting neuronal frequency tuning and selectivity. At present, a detailed understanding of the relation between tone response properties of AC neurons and frequency discrimination behavior remains missing.

Neurons in AC form mutually coupled excitatory–inhibitory networks, which shape the responses of neurons to sounds [32,33]. Electrophysiological recordings and pharmacological studies demonstrate that GABA-ergic inhibition controls tone-evoked response amplitude, spontaneous firing rate and frequency selectivity [25,27], among other aspects of excitatory neuronal responses. The most common type of interneurons, parvalbumin-positive interneurons (PVs) [11,34–38], which target the pyramidal cell bodies, gate feed-forward thalamocortical auditory inputs [38]. We postulated that optogenetically modulating PV activity would affect tone-evoked responses in the AC, thereby enabling us to examine the effect of changing tone response properties of AC neurons on auditory behavior and learning. We focused on two behaviors, frequency discrimination—driven prepulse inhibition (PPI) of the acoustic startle response (ASR), and differential auditory fear conditioning (DAFC). Frequency discrimination-driven PPI relies on an innate behavior—startle response to loud noise, and is controlled by subcortical circuits [39]. Because PPI decreases if the startle noise is preceded by a change in an acoustic stimulus, it can be used to measure frequency discrimination acuity [3,40]. By contrast, DAFC requires both learning and memory and is controlled by interactions between the cortex and a complex circuit including the amygdala and the hippocampus [41–44]. While these two behaviors rely on different brain circuits, they can affect each other [45], with the AC facilitating this interaction [3].

We found that cortical inhibition controls frequency discrimination acuity and frequency specificity of auditory fear conditioning. These behavioral changes were correlated with changes in the magnitude of tone-evoked neuronal activity.

## Results

### Effective and Specific Optogenetic Modulation of Neuronal Activity in AC

**Fig 1:**
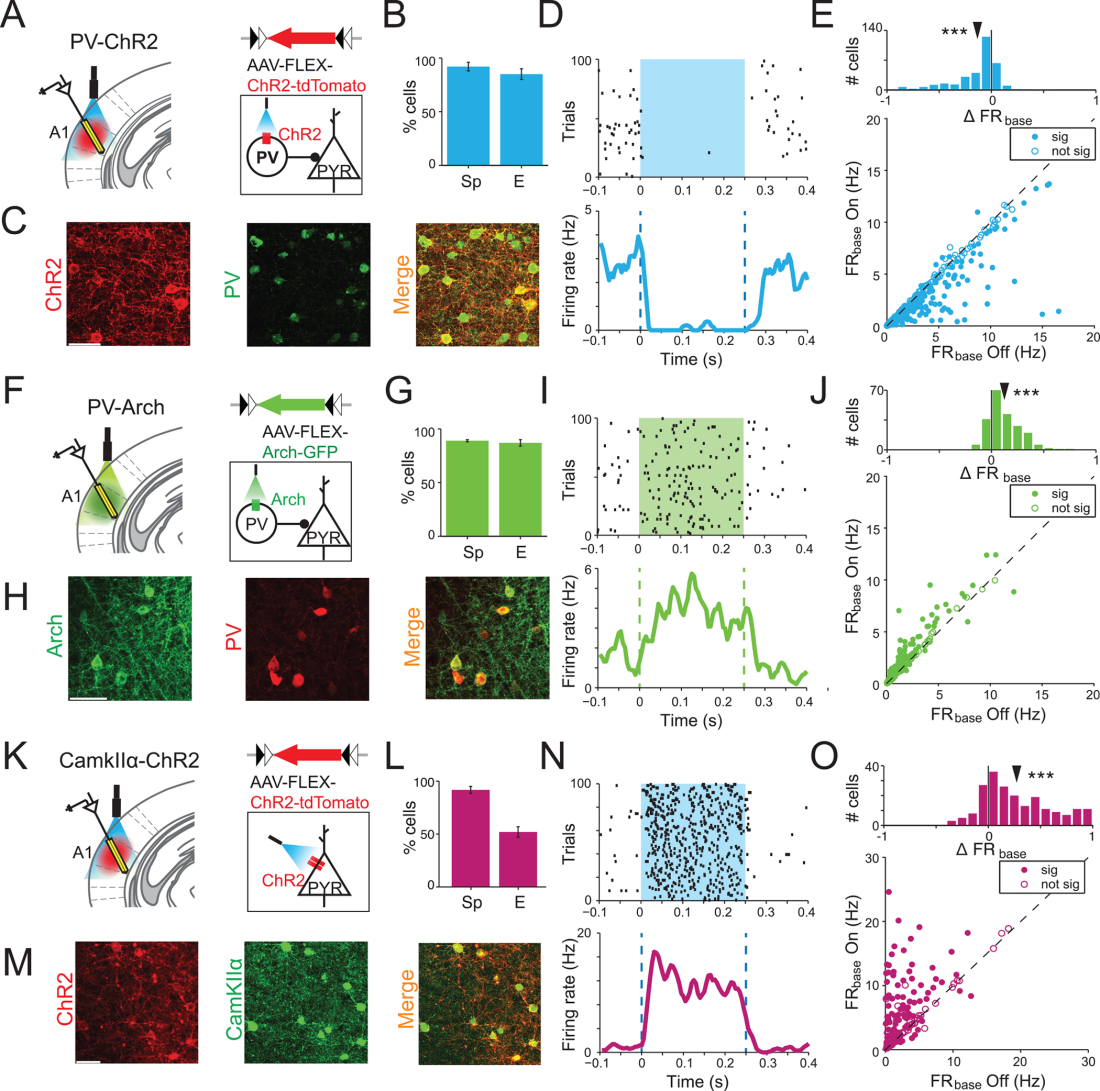
Optogenetic silencing or activation of neuronal activity. A, F, K. PV-Cre mice were injected bilaterally with either AAV-FLEX-ChR2-tdTomato (A) or AAV-FLEX-Arch-GFP (F). CamKIIα-Cre mice (K) were injected with AAV-FLEX-ChR2-tdTomato. All animals were implanted with optical fibers bilaterally targeting AC and neuronal activity was recorded using a multichannel silicon probe in AC (left panel). Schematic of the circuits targeted by photomodulation (right panel). PV-ChR2 group: Blue light (473 nm) activates PVs, thereby inhibiting excitatory neurons in mice expressing ChR2 in PVs. PV-Arch group: Green light (532 nm) suppresses PVs, thereby activating excitatory neurons in mice expressing Arch in PVs. CamKIIα-ChR2 group: Blue light directly activates excitatory neurons in mice expressing ChR2 in excitatory neurons. B, G, L. Specificity (Sp) and effectiveness (E) of viral expression in PV-ChR2 group (B, *n* = 4 mice), PV-Arch group (G, *n* = 3), and CamKIIα-ChR2 group (L, *n* = 2). Bars represent mean ± SEM (standard error of the mean). C, H, M. Immunohistochemistry demonstrating coexpression of the virus with the respective cell type in AC. A.: Channelrhodopsin-tdTomato (ChR2-tdTomato) expressed in PV-Cre mouse AC. Red: tdTomato. Green: antibody for parvalbumin. H.: Archaerhodopsin-GFP (Arch-GFP) expressed in PV-Cre mouse AC. Green: GFP. Red: antibody for parvalbumin. M.: ChR2-tdTomato expressed in CamKIIα-Cre mouse AC. Red: tdTomato. Green: antibody for CamKIIα. Scale bar, 50 μm. D, I, N. Responses of neurons to optogenetic stimulation. Light was presented from 0 to 0.25 s (color rectangle). Top: Raster plot of spike times of a representative neuron activated by photostimulation from each of PV-ChR2 (D), PV-Arch (I), and CamKIIα-ChR2 (N) group. Bottom. Corresponding peristimulus time histogram (PSTH) of neuronal response in light-On (color) and light-Off (gray) conditions. E, J. Optogenetic activation of PVs in PV-ChR2 group (E) suppressed spontaneous firing rate (FR_base_), whereas suppression of PVs in PV-Arch mice (J) increased FR_base_ of neurons recorded from AC. Bottom: Scatter plot of spontaneous firing rate on light-On plotted versus light-Off trials. Each circle represents a single unit. Closed and open circles represent significant and nonsignificant effect of light respectively (paired *t* test, comparing FR 50 ms before and after light onset). Top: Histograms of index of change in FR_base_ due to photoactivation (E) and photosuppression (J) of PVs over the neuronal population. Arrowhead: mean. (PV-ChR2: ΔFR_base_ = −0.13; PV-Arch: ΔFR_base_ = 0.12); ***: *p* < 0.001 (one-sample *t* test; PV-ChR2: *n* = 330, *t*_329_ = 11.2, *p* = 7.4e-25; PV-Arch: *n* = 212, *t*_211_ = 11.8, *p* = 4.9e-25). See data in S1 Data. O. Direct photoactivation of CamKIIα neurons leads to a significant increase in FR_base_. Top: index of change in the FR_base_ across neuronal population. Bottom: FR_base_ in light-On trials versus light-Off trials. ***: one-sample *t* test, *n* = 206, t_205_ = 11.84, *p* = 5.4e-25, mean ΔFR_base_ = 0.27. See data in S1 Data.

To manipulate the level of activity of a specific type of inhibitory interneuron, PVs, in AC, we drove them to express Channelrhodopsin (ChR2) or Archaerhodopsin (Arch), using targeted viral delivery to AC (Fig 1A and 1F) [34,38,46]. Arch is a light-driven proton pump that hyperpolarizes the neuron when activated with green light [35]. Conversely, ChR2 is a light-gated cation channel that depolarizes the neuron when activated with blue light [47]. We injected a modified adeno-associated virus (AAV), which carried the antisense code for either opsin under the FLEX cassette in AC of PV-Cre mice. Following an incubation period, PVs in AC expressed ChR2 or Arch efficiently and with high specificity (Fig 1B and 1C and Fig 1G and 1H). Analysis of light-evoked responses of putative PVs showed that PVs have a distinct waveform with relatively deep troughs (S1 Fig). We used spike waveform shape as a criterion for exclusion of putative PVs from the pool of analyzed neurons.

Throughout the study, we compared the effects of interneuron modulation with that of direct increase in the activity of excitatory neurons by photostimulation. This control allowed us to test whether a simple elevation of the activity level of excitatory neurons can account for the observed results. In order to activate excitatory neurons directly, we drove them to express ChR2 in AC using targeted viral delivery in mice that express Cre recombinase in neurons under CamKIIα promoter. This resulted in efficient and specific expression of ChR2 in putative excitatory neurons in AC (Fig 1K–1M).

To verify the effectiveness of optogenetic modulation, we measured the effect of the laser on the spontaneous firing rate of AC neurons. Spiking activity of neurons in AC of awake, head-fixed mice was recorded during acoustic presentation of a random tone sequence, a stimulus designed to measure the frequency tuning curve of neurons. Locally shining either blue (473 nm) or green light (532 nm) suppressed or activated the activity of putative excitatory neurons confined to AC, respectively (S2 Fig). Activation of PVs (473 nm, 0.2 mW/mm^2^ intensity at the fiber tip) significantly reduced the spontaneous firing rate (FR_base,_ computed during the baseline period, 0–50 ms prior to tone onset) in a large fraction of recorded neurons (Fig 1D and 1E), resulting in a reduced mean spontaneous firing rate over the recorded neuronal population. The effect scaled with increasing light intensity: the index of change in FR_base_ increased with increased activation of ChR2 (S3A and S3B Fig). Conversely, suppression of PVs (532 nm, 10 mW/mm^2^) increased mean FR_base_ (Fig 1I and 1J). These changes in spontaneous firing rate demonstrate that the optogenetic manipulation of PV activity efficiently altered neuronal activity in the AC.

Direct optogenetic manipulation of excitatory neurons was similarly effective: photoactivation of CamKIIα neurons by blue light increased the spontaneous activity of neurons (Fig 1N and 1O). The effect of light in the CAMKIIα-ChR2 group was either the same or larger than in the PV-Arch group (S2 Fig), allowing for comparison of effects of suppression of PVs in PV-Arch group to direct activation of excitatory neurons in CAMKIIα-ChR2 group.

### Modulating Interneuron Activity in AC Leads to Changes in Behavioral Frequency Discrimination Acuity

**Fig 2:**
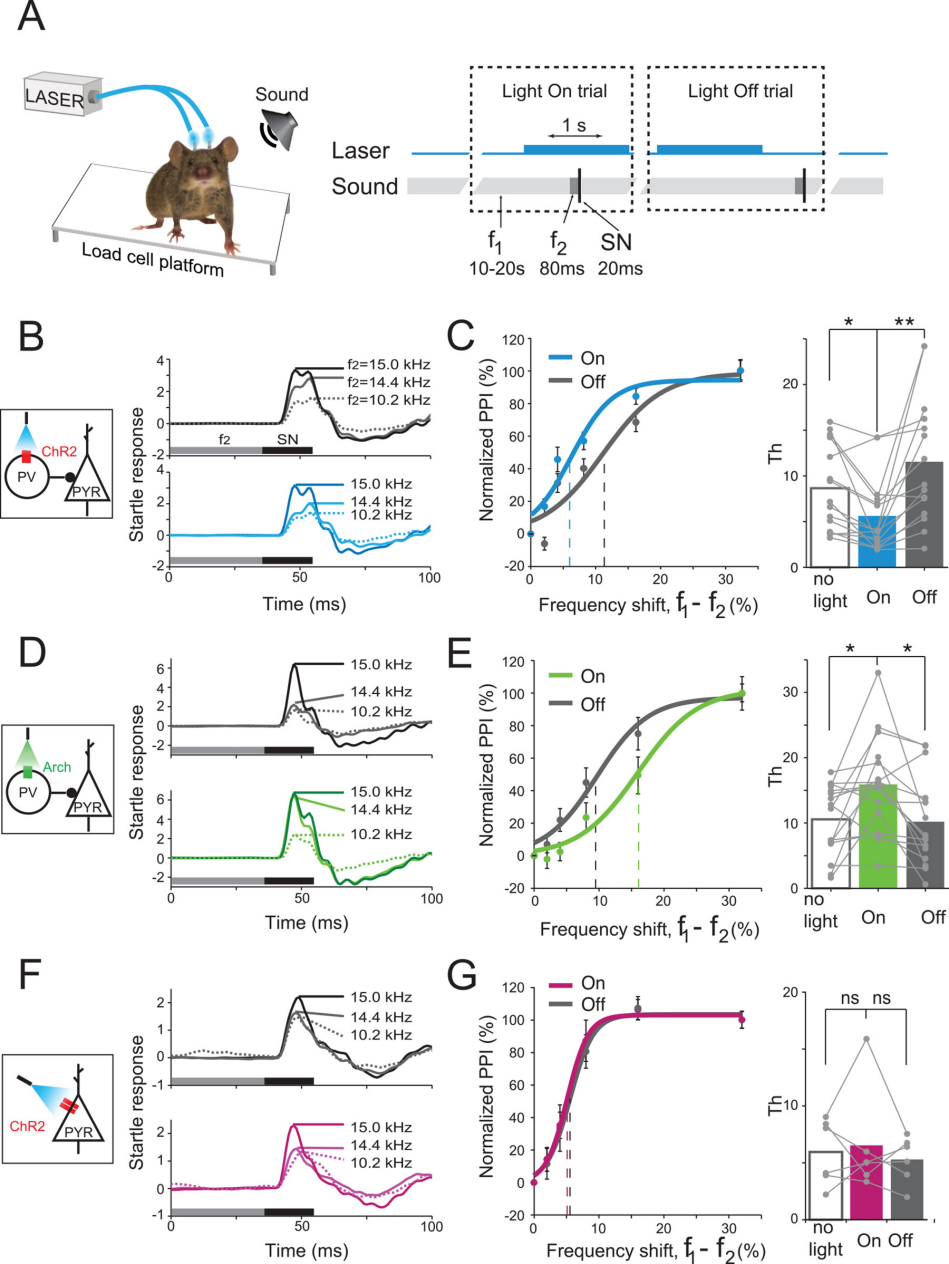
Cortical inhibitory neurons bidirectionally regulate frequency discrimination acuity. A. Experimental design. On each trial, a sequence of three acoustic stimuli was presented: background tone (f_1_, 15 kHz, 10–20 s), prepulse tone (f_2_, 10.2–15 kHz, 80 ms), and startle broadband noise (SN, 20 ms). In light-On trials, the laser (1 s, blue bar) was activated overlapping with the prepulse. In light-Off trials, the laser did not overlap with the prepulse. B, D, F. Left. Diagram shows circuits targeted by photomodulation. Right. Representative examples of the ASR (pressure applied by the mouse on the load cell platform in responses to startle noise) in PV-ChR2 (B), PV-Arch (D), and CamKIIα-ChR2 (F) mice. Top. Mean ASR for 10 light-Off trials in one session. Bottom. Mean ASR for 10 light-On trials in the same session. Note that ASRs decrease as the frequency shift between 15 kHz background tone and prepulse tone (f_2_) increases. C, E, G. Left. PPI as a function of frequency shift between the prepulse and the background tone on light-On (color) and light-Off (gray) trials. Vertical dashed lines: *Th*. Error bars: Mean ± SEM. Right. *Th* threshold for light-On and light-Off trials and for separate “no light” session, in which no photostimulation was presented. C. Photoactivation of PVs in PV-ChR2 group decreased *Th* (paired t-test with Bonferroni adjustment for comparison between performance on "light-on" trials to "no-light" session and "light-off" trials, *t*_14_ = 3.2, *p* = 0.01; *t*_14_ = 3.6, *p* = 0.006; *n* = 15 mice). E. Photosupression of PVs in PV-Arch group increased *Th* PV-Arch group (t_15_ = 2.6, *p* = 0.034; *t*_15_ = 3.2, *p* = 0.012; *n* = 16). G. Increasing activity level of excitatory neurons in CamKIIα-ChR2 mice did not affect behavioral *Th*. ns: paired *t* test, *n* = 6, *t*_5_ = 0.78, *p* = 0.47; *t*_5_ = 0.36, *p* = 0.73. Dots depict data for an individual subject. Bars depict mean value for each group. See data in S1 Data.

We next tested the function of PVs in behavioral frequency discrimination acuity. To determine whether PV activity affects behavioral frequency discrimination acuity, we measured the change in frequency discrimination threshold (*Th*), while modulating PV activity. *Th* was determined by measuring the percent inhibition of the ASR due to a shift in frequency between a background and a pre-pulse tone for varying frequencies of the pre-pulse [3,40] (Fig 2A). Strong PPI of the ASR indicates that the mouse detected the shift in frequency between the background and prepulse tones (Fig 2B, 2D and 2F). As previously reported [3], PPI increased with larger frequency shifts between the background and prepulse tones. This method thus provides psychometric response curves for frequency discrimination over the course of a single session that lasts less than 1 hr and does not require training the subject. *Th* was computed as the percent difference in frequency between the background and the prepulse tone that elicited 50% of the maximum PPI (S4 Fig). To test the effect of PV activity on *Th*, the laser was turned on during half of the behavioral trials, overlapping with the startle and prepulse stimuli (light-On trial). On the remaining (light-Off) trials, the laser was turned on at a quasirandom time during intertrial interval. In an additional test session, the light was not used throughout (no-light).

Optogenetic modulation of PV activity significantly affected behavioral frequency discrimination acuity. Activating PVs improved frequency discrimination acuity, as evidenced by a reduction in *Th* for light-On trials as compared to light-Off trials and no-light session in PV-ChR2 mice (Fig 2C). Suppressing PV activity reduced frequency discrimination acuity, leading to a significant increase in *Th* in PV-Arch mice (Fig 2E). Combined, these results demonstrate that the level of PV activity bidirectionally controls behavioral frequency discrimination acuity.

We performed several controls to ensure that the effects of photomodulation of PV activity were specific to the shift in frequency and could not be explained by a change in the ability of the mouse to respond to and to hear the stimuli. First, we tested whether light alone affected *Th*. In a control group of PV-Cre mice, in which PVs were driven to express only the fluorescent marker, but not the opsin, light did not affect *Th* (S5 Fig). This indicates that the observed change in *Th* required the expression of opsins in PVs. In mice expressing ChR2 or Arch, neither activation nor suppression of PVs affected the magnitude of ASR elicited by startle stimulus alone (S6A Fig). Therefore, the observed change in *Th* was not simply due to a change in the magnitude of ASR. Furthermore, activating or suppressing PVs did not lead to a change in the maximum PPI elicited by the pre-pulse tone (S6B Fig). These tests indicate that photomodulation of PV activity did not affect the ability of the mouse to detect large shifts in frequencies.

To further test that photomodulation of PVs did not impair the mouse's ability to hear test tones, we measured PPI due to the prepulse tones alone, without the background, as an estimate of how strongly the mouse could detect the prepulse tone. PPI elicited by the pre-pulse tones was not significantly different on light-On and light-Off trials (S7 Fig). Furthermore, there was no difference in PPI elicited by the prepulse tone at all six frequencies tested, indicating that mice detected the different tones similarly well on both light-On and light-Off trials. Taken together, these controls demonstrate that the observed change in *Th* cannot be explained by changes in more basic aspects of mouse hearing or a non-specific effect of photostimulation.

We repeated the experiments, activating the excitatory neurons directly in the CamKIIα-ChR2 group. In striking contrast to the effect of PV inactivation in PV-Arch mice, direct activation of principal neurons did not affect *Th* (Fig 2G). This result demonstrates that the change in *Th* due to photosuppresson of PV activity is specific to the effect of inhibitory interneurons, and is not simply due to an increase in the mean firing rate of excitatory neurons during PV suppression.

### Changes in Behavioral Frequency Discrimination Acuity due to Photomodulation of PVs Are Correlated with Changes in the Neuronal Tone-Evoked Response Magnitude

**Fig 3:**
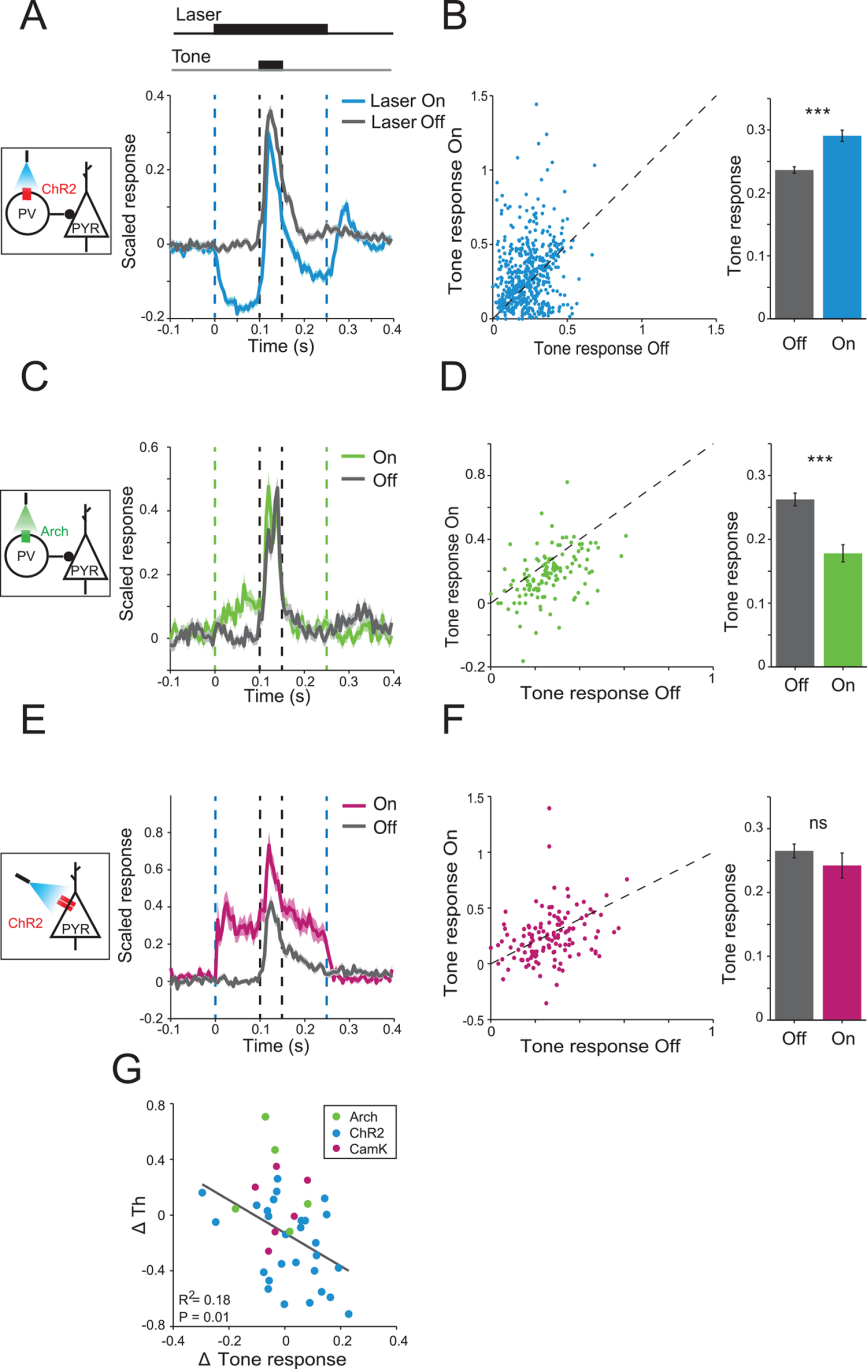
Activating PVs increases tone-evoked responses, whereas suppressing PVs has the opposite effect. A, C, E. Scaled time course of the firing rate of the neurons in response to a tone (outlined by black dashed lines) on light-On (color) and light-Off (gray) trials. Time of laser onset and offset is outlines by vertical color dashed lines. Mean ± SEM. A. PV-ChR2 mice. C. PV-Arch mice. E. CamKIIα-ChR2 mice. Inset diagram shows circuits targeted by photomodulation. B, D, F. Left. Scaled responses to tones on light-On trials plotted against responses on light-Off trials for putative excitatory neurons. Response magnitude is defined as a difference in mean scaled FR_base_ (0–50 ms before tone onset) and mean response to tone (FR_tone_, 0–50 ms after tone onset). Right. Mean ± SEM. responses to tones from the left panel. See data in S1 Data. B. PV-ChR2 mice: Tone-evoked responses on light-On trials (blue) were significantly higher than on light-Off trials (gray). Paired *t* test, *n* = 550, *t*_549_ = 5.81, *p* = 1.1e-8. Data are combined for three laser powers used to activate PV interneurons (0.2, 0.5, and 10 mW/mm^2^). D. PV-Arch mice: Tone-evoked responses on light-On trials (green) were significantly lower than on light-Off trials (gray). Paired *t* test, *n* = 127, *t*_126_ = 6.70, *p* = 6.3e-10. F. CamKIIα-ChR2 mice: Tone-evoked responses were not significantly affected by light. Paired *t* test, *n* = 130, *t*_129_ = 1.19, *p* = 0.22. G. Change in the magnitude of scaled response to tones is correlated with change in behavioral *Th* due to manipulation of PVs activity. Each dot represents data averaged for single units from each subject at one light intensity (only subjects with >5 identified single units were included). Blue: PV-ChR2 group (*n* = 28); Green: PV-Arch group (*n* = 5). Magenta: CamkIIα-ChR2 group (*n* = 6, not included in regression analysis). *p* = 0.01.

Can the changes in neuronal activity in AC evoked by the different types of optogenetic manipulation explain the behavioral results? To answer this question, we measured how strongly photostimulation of PVs affected the responses of neurons during tone presentation in a frequency band of one octave centered at the best frequency (BF). To estimate the relative strength of population neuronal responses to tones, we computed the tone-evoked response magnitude measured as a difference between mean firing rate during tone presentation (FR_tone_) and FR_base_ (Fig 3). Photomodulation of PVs resulted in a significant change in the magnitude of normalized tone-evoked response over the population of putative excitatory neurons. Photoactivation of PVs increased the tone-evoked response magnitude (Fig 3A and 3B). This effect was due to a relatively weaker decrease in FR_tone_ as compared to the decrease in FR_base_ evoked by PV photoactivation (Fig 3A and S3 Fig). By contrast, photosuppression of PVs led to a decrease in tone-evoked response magnitude (Fig 3C and 3D). This effect was due to a relatively weaker increase in FR_tone_ as compared to FR_base_ (Fig 3C). These results were consistent with the mean behavioral results for changes in *Th*: PV photoactivation, which improved behavioral frequency discrimination acuity, also increased mean tone-evoked responses; whereas PV photosuppression, which impaired behavioral frequency discrimination acuity, also suppressed mean tone-evoked responses in AC.

The effects of PV inactivation differed between subjects. Therefore, we computed a correlation between neuronal responses and behavioral performance over subjects by comparing the mean tone-evoked response magnitude over all neurons and behavioral *Th* for each mouse. Changes in neuronal responses caused by photomodulation of PV activity were significantly inversely correlated with changes in *Th* measured behaviorally (Fig 3G). This correlation suggests that the measured change in magnitude of tone-evoked responses in AC is a good predictor for the change in behaviorally measured frequency discrimination acuity.

By contrast, direct photoactivation of excitatory neurons in the CamKIIα-ChR2 group did not affect the tone-evoked response magnitude (Fig 3E and 3F). This result is due to the strong increase in both the spontaneous and tone-evoked activity of recorded neurons by direct photoactivation of excitatory neurons (S8A and S8B Fig). These results are consistent with the lack of change in behavioral frequency discrimination acuity due to photoactivation of excitatory neurons.

Combined, our findings support the interpretation that both the bidirectional modulation of *Th* due to PV stimulation, and the lack of modulation due to excitatory neuronal stimulation, are due to changes in the magnitude of tone-evoked responses relative to the baseline firing rate of AC neurons.

### Changes in Behavioral Frequency Discrimination Acuity due to Photomodulation of PVs Are Not Consistently Correlated with Changes in Neuronal Frequency Selectivity

**Fig 4:**
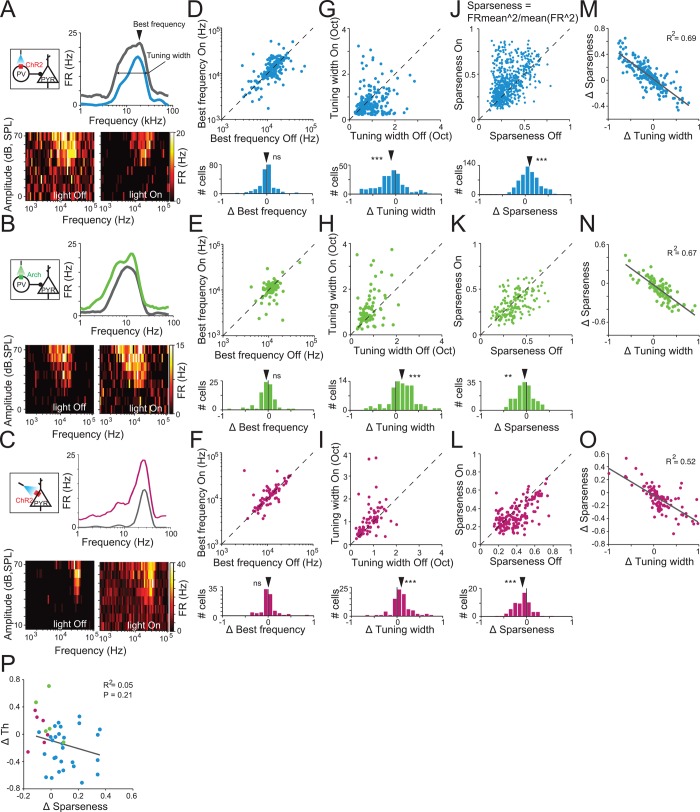
Modulating PV activity does not affect frequency tuning, but bidirectionally affects frequency selectivity. A, B, C. Frequency response function (top) and tuning curve (bottom) of a putative excitatory neuron in the absence of photostimulation (light-Off trials) and during photostimulation of AC(light-On trials). Inset diagram shows circuits targeted by photomodulation. A. PV-ChR2: light activates PVs. B. PV-Arch: light suppresses PVs. C. CamKIIα-ChR2: light activates excitatory neurons. D, E, F. Scatter plot (top) shows distribution of the BF for putative excitatory neurons in light-On and light-Off trials. Histogram (bottom) shows index of change in the BF due to photostimulation. See data in S1 Data. D. PV-ChR2 group: Photoactivation of PVs had no significant effect on the BF of the frequency response function. One-sample *t* test. *n* = 233, mean ΔBF = −0.01, t_232_ = 0.94, *p* = 0.35. E. PV-Arch group: Photosuppression of PVs did not significantly affect the BF of the frequency response function. One-sample *t* test. *n* = 83, mean ΔBF = −0.04, t_82_ = 1.98, *p* = 0.051. F. CamKIIα-ChR2 group: Direct photoactivation of excitatory neurons did not significantly affect the BF of the frequency response function. One-sample *t* test. *n* = 82, mean ΔBF = −0.004, t_81_ = 0.22, *p* = 0.82. G, H, I. Scatter plot (top) shows distribution of the tuning width for putative excitatory neurons in light-On and light-Off trials. Histogram (bottom) shows index of change in the tuning width due to photostimulation. See data in S1 Data. G. PV-ChR2 group: Photoactivation of PVs significantly decreased the tuning width of the frequency response function. One-sample *t* test, mean ΔBW = −0.10, *t*_232_ = 5.17, *p* = 5.2e-7. H. PV-Arch group. Photosuppression of PVs significantly increased the tuning width of the frequency response function. One-sample *t* test mean ΔBW = 0.13, *t*_82_ = 4.31, *p* = 4.5e-5. I. CamKIIα-ChR2 group. Direct photoactivation of excitatory neurons significantly increased the tuning width of the frequency response function. One-sample *t* test mean ΔBW = 0.09, *t*_81_ = 3.77, *p* = 4.5e-5. J, K, L. Scatter plot (top) shows distribution of sparseness for putative excitatory neurons in light-On and light-Off trials. Histogram (bottom) shows index of change in sparseness due to photostimulation of PVs. J. PV-ChR2 group: Photoactivation of PVs led to an increase in sparseness of the frequency response function. One-sample *t* test, mean ΔSparseness = −0.09, *t*_631_ = 11.0, *p* = 6.6e-26. K. PV-Arch group. Photosuppression of PVs led to a decrease in sparseness. One-sample *t* test mean ΔSparseness = −0.04, *t*_158_ = 2.96, *p* = 0.04. L. CamKIIα-ChR2 group. Direct photoactivation of excitatory neurons led to a decrease in sparseness. One-sample *t* test, mean ΔSparseness = −0.09, *t*_151_ = 6.01, *p* = 1.3e-8. M–O. Change in sparseness due to photostimulation was negatively correlated with the change in tuning width in all tested groups: PV-ChR2 (M, *p* = 7.4e-61), PV-Arch (N, *p* = 2.7e-21), CamKIIα-ChR2 (O, *p* = 6.3e-19). P. Change in sparseness did not significantly correlate with behavioral *Th* due to manipulation of PVs activity. Each dot represents data averaged for single units from each subject at one light intensity (only subjects with >5 identified single units were included). Blue: PV-ChR2 group (*n* = 28); Green: PV-Arch group (*n* = 5). Magenta: CamKIIα-ChR2 group (*n* = 6, not included in regression analysis). *p* = 0.21. For PV-ChR2 mice, data are combined over three laser powers used to activate PV interneurons (0.2, 0.5, and 10 mW/mm^2^). J, K, L Data for putative excitatory neurons that showed increased FR in response to tones (“auditory” neurons). PV-ChR2: *n* = 632; PV-Arch: *n* = 159; CamKIIα-ChR2: *n* = 152. D–I, M–O. Data for “auditory” neurons fitted to Gaussian function at *R* ^*2*^ > 0.4. PV-ChR2: *n* = 233; PV-Arch: *n* = 83, CamKIIα-ChR2: *n* = 82.

Frequency discrimination may be controlled not only by the firing rate of neurons but also by their frequency tuning properties [48]. Therefore, we next quantified the effect of PV photo-modulation on the frequency tuning properties of putative excitatory neurons. The mean firing rate of neuronal responses to tones was used to construct a tuning curve for the frequency and intensity level of the tones, computed on light-Off and light-On trials, separately (Fig 4A–4C).

It has previously been suggested that excitatory and inhibitory inputs to the same neurons exhibit similar frequency tuning properties in AC [49]. We therefore expected that the BF (the frequency of the tone eliciting the highest firing rate) would not be affected by PV photostimulation. Indeed, the BF of recorded units was not affected by PV photoactivation and photosuppression (Fig 4D and 4E, respectively). However, PVs exhibit tuning that is similar [50] or lower [51] in selectivity to excitatory neurons. Therefore, manipulation of PV activity would likely affect the frequency selectivity of putative excitatory neurons to tones. Indeed, photostimulation modulated frequency selectivity of neuronal responses. We quantified frequency selectivity by two measures: the width of frequency tuning and the sparseness of the frequency response function. Tuning width was computed as twice the standard deviation of the Gaussian fit to the frequency response function. Tuning width decreased during activation of PVs and increased during suppression of PVs (Fig 4G and 4H, respectively). We used sparseness as an additional measure for frequency tuning selectivity, because it is less sensitive to the magnitude of the firing rate as well as spontaneous firing rate than tuning width. In addition, sparseness does not assume a specific shape of the frequency response function. A sparseness value of 1 indicates that the neuron responds to tone at only one frequency, whereas a sparseness value of 0 indicates that the neuron responds equally strongly to tones at all frequencies. Activating PVs significantly increased sparseness over the population of putative excitatory neurons (Fig 4J). The strength of the effect of photoactivation on neuronal sparseness increased with light intensity (S9A and S9B Fig) and was significantly correlated with the change in the baseline firing rate (S9C Fig). Conversely, suppressing the activity of PV interneurons significantly reduced the sparseness of neuronal responses to tones (Fig 4K). As expected, the effects of photomodulation on sparseness and tuning width were significantly correlated (Fig 4M and 4N). Combined, we found that up- or down-regulating activity of PVs did not affect the BF of neurons, but modulated the tuning selectivity of principal AC neurons, such that activating PVs increased neuronal frequency selectivity, whereas suppressing PVs reduced neuronal frequency selectivity.

On average, the mean changes in frequency selectivity were consistent with behavioral results: activation of PVs, which improved frequency discrimination acuity, increased frequency selectivity in AC neurons, whereas suppression of PVs, which impaired frequency discrimination acuity, decreased frequency selectivity in AC. However, when examined on an animal-by-animal level, there was no significant correlation between frequency sparseness and the change in behavioral threshold when tested using either parametric or nonparametric tests (Fig 4P). This result suggests that mean frequency selectivity may not be as important for behavioral frequency discrimination acuity as the response magnitude for tones of preferred frequencies.

We next tested whether photoactivation of excitatory neurons affected mean neuronal frequency tuning. Over the population of recorded neurons, the BF was not affected (Fig 4F). However, the tuning width increased significantly (Fig 4I), whereas sparseness of frequency responses decreased (Fig 4L). As in PV-Cre mice, the tuning width and sparseness significantly correlated with each other (Fig 4O). These measurements contrast with the behavioral findings that photoactivation of excitatory neurons does not affect frequency discrimination acuity, further supporting the interpretation that frequency selectivity may not be as important for behavioral frequency as changes in tone-evoked response magnitude.

### Modulation of PV Activity Level in AC Leads to Changes in Specificity of Auditory Fear Conditioning

**Fig 5:**
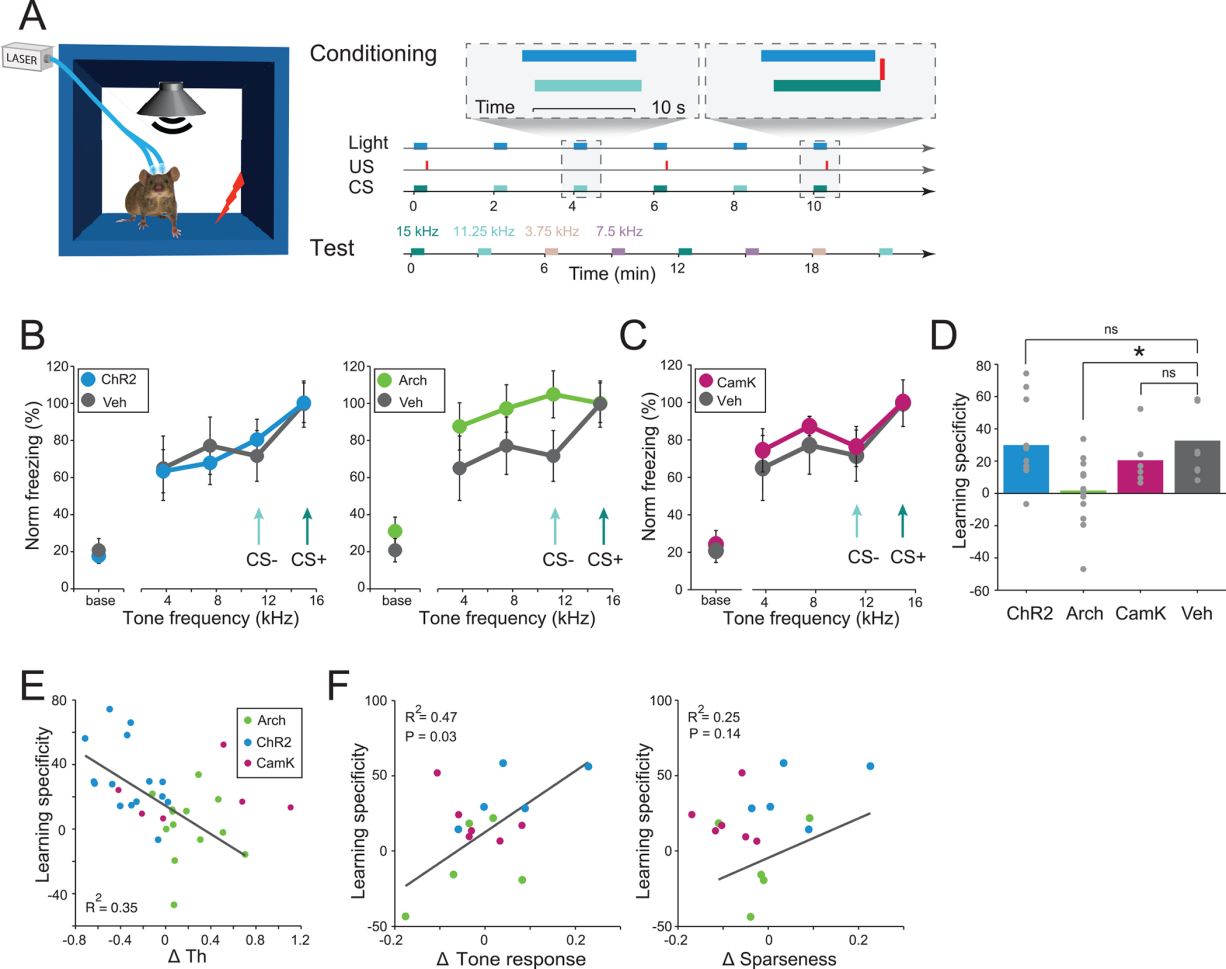
PV neurons control learned frequency specificity. A. Diagram of DAFC and testing protocols. During DAFC, both CS+ and CS− overlapped with photostimulation of the AC by blue (PV-ChR2 group) or green light (PV-Arch group). During the test session 24 h later, tones at 4 frequencies were presented without US and photostimulation. B. Results of the test for LS in PV-ChR2 (left, *n* = 13) and PV-Arch (right, *n* = 15). PV-Arch group (green) showed no significant decline in freezing to test tones (repeated measures ANOVA, *F*_3,42_ = 0.92, *p* = 0.44), whereas PV-ChR2 group (blue) and control group (gray, *n* = 8) showed significant decline in freezing (*F*_3,36_ = 15.5, *p* < 0.0001; *F*_3,21_ = 4.17, *p* = 0.018 respectively). Mean ± SEM. Arrows depict frequencies used as CS− and CS+ during conditioning. C. LS test for CamKIIα-ChR2 (*n* = 6, magenta) and control group (*n* = 8, gray). Mean ± SEM. CamKIIα-ChR2 mice showed significant decline in freezing (repeated measures ANOVA *F*_3,15_ = 5.83, *p* = 0.008). Arrows depict frequencies used as CS− and CS+ during conditioning. D. Average LS index (LS) for mice in PV-Arch group (green bar) was significantly lower than LS for mice in the control group injected with a control viral construct (gray bar, *t* test with Bonferroni adjustment, *t*_19_ = 3.28, *p* = 0.012). Mean LS for PV-ChR2 (blue) and CamKIIα-ChR2 group (magenta) were not significantly different from LS for control mice. ns: *t* test, t_21_ = 0.1, *p* = 0.92 and *t*_12_ = 1.14, *p* = 0.28 respectively. Dots depict data for an individual subject. Bars depict mean value for each group. E. Specificity of the freezing response versus index of change in *Th* due to photostimulation of PV activity. Each circle depicts a single mouse. Green: PV-Arch group (*n* = 13 mice). Blue: PV-ChR2 group (*n* = 15). Pearson = 0.59, R^2^ = 0.35, *p* = 0.0009. CamKIIα-ChR2 group (*n* = 6) is shown in magenta but not included in statistical analysis. See data in S1 Data. F. Specificity of freezing responses (left) but not sparseness (right) significantly correlated with the change in the magnitude of tone-evoked responses. Green: PV-Arch group (*n* = 5). Blue: PV-ChR2 group (*n* = 5). Magenta: CamKIIα-ChR2 group (*n* = 6) is not included in correlation analysis. See data in S1 Data.

Thus far in the behavioral test, we examined frequency discrimination acuity using a modified procedure that relied on measuring inhibition of the startle response by a tone preceding the startle noise—an innate behavioral response measured as PPI. We then tested whether inhibition in AC also modulated auditory associative learning [52]. In DAFC, the mouse is presented with two tones of different frequencies, one of which (CS+) is associated with an aversive stimulus (mild electric foot shock) and one that is not (CS−) (Fig 5A and S10 Fig). 24 h later, the mice typically exhibit an increase in conditioned response (freezing) during presentation of CS+ and a smaller increase in freezing during presentation of CS−. For different subjects, the freezing response may be specific to CS+ or generalize to tones at frequencies beyond CS− [3]. We hypothesized that specificity of freezing after conditioning may be controlled by PVs in AC. To test this hypothesis, we measured whether up- or down-regulating the activity of PVs in AC during conditioning affects the specificity of the freezing response.

We subjected four groups of mice to DAFC, overlapping light and tone presentation. In the PV-Arch group, suppression of PVs during conditioning led to activation of putative excitatory neurons. In the PV-ChR2 group, photoactivation of PVs during conditioning led to suppression of putative excitatory neurons. In the control group of mice, which were injected with control vector that encoded only fluorescent protein, PVs were not affected by the laser. In CamKIIα-ChR2 group, the activity of excitatory neurons was enhanced during conditioning. 24 h following DAFC, we tested the level of freezing to CS+, CS− and two additional tones during the LS test, designed to measure how specific freezing response was to conditioned tones (Fig 5A). We then assessed the level of specificity of conditioned response by measuring the relative difference in freezing response to the CS+ tone and mean freezing response to test tones (LS index, Methods).

In all groups, mice exhibited an increase in the freezing response to CS+ (Fig 5B and 5C). However, mice in which PVs were suppressed during conditioning did not exhibit differential freezing response to CS+ and CS−. By contrast, mice in both the PV-ChR2 and the control groups exhibited a significant reduction in freezing to CS− as compared to CS+. Furthermore, the specificity of learned freezing as measured by LS was significantly lower than for mice in PV-Arch group than for mice in control group (Fig 5D). Interestingly, direct activation of excitatory neurons in CamKIIα-ChR2 group did not result in significant change of LS (Fig 5C and 5D). Thus, we find that suppressing PV activity during conditioning led to a decrease in specificity of auditory fear conditioning, whereas either increasing PV activity or increasing the general level of activity of excitatory neurons did not have a significant effect on the specificity.

As expected, between subjects, the level of specificity of conditioned fear varied. If inhibition in AC controls both the frequency discrimination acuity and specificity of the conditioned response via a similar mechanism, we expected the behavioral measures for acuity and specificity to be correlated. Indeed, change in behavioral frequency discrimination acuity due to photostimulation was significantly correlated with the change in specificity of auditory fear conditioning (Fig 5E). Furthermore, there was a significant correlation between LS and the effect of photomodulation of PVs activity on neuronal tone-evoked response magnitude but not sparseness (Fig 5F). Combined, these findings demonstrate that neuronal response magnitude in AC regulates not only behavioral frequency discrimination acuity measured through a test of innate behavior, but also specificity of associative learning.

### Mutually Coupled Excitatory-Inhibitory Neuronal Model Accounts for Differential Effects of PV and Excitatory Neuronal Modulation on Tone-Evoked Response Magnitude

**Fig 6:**
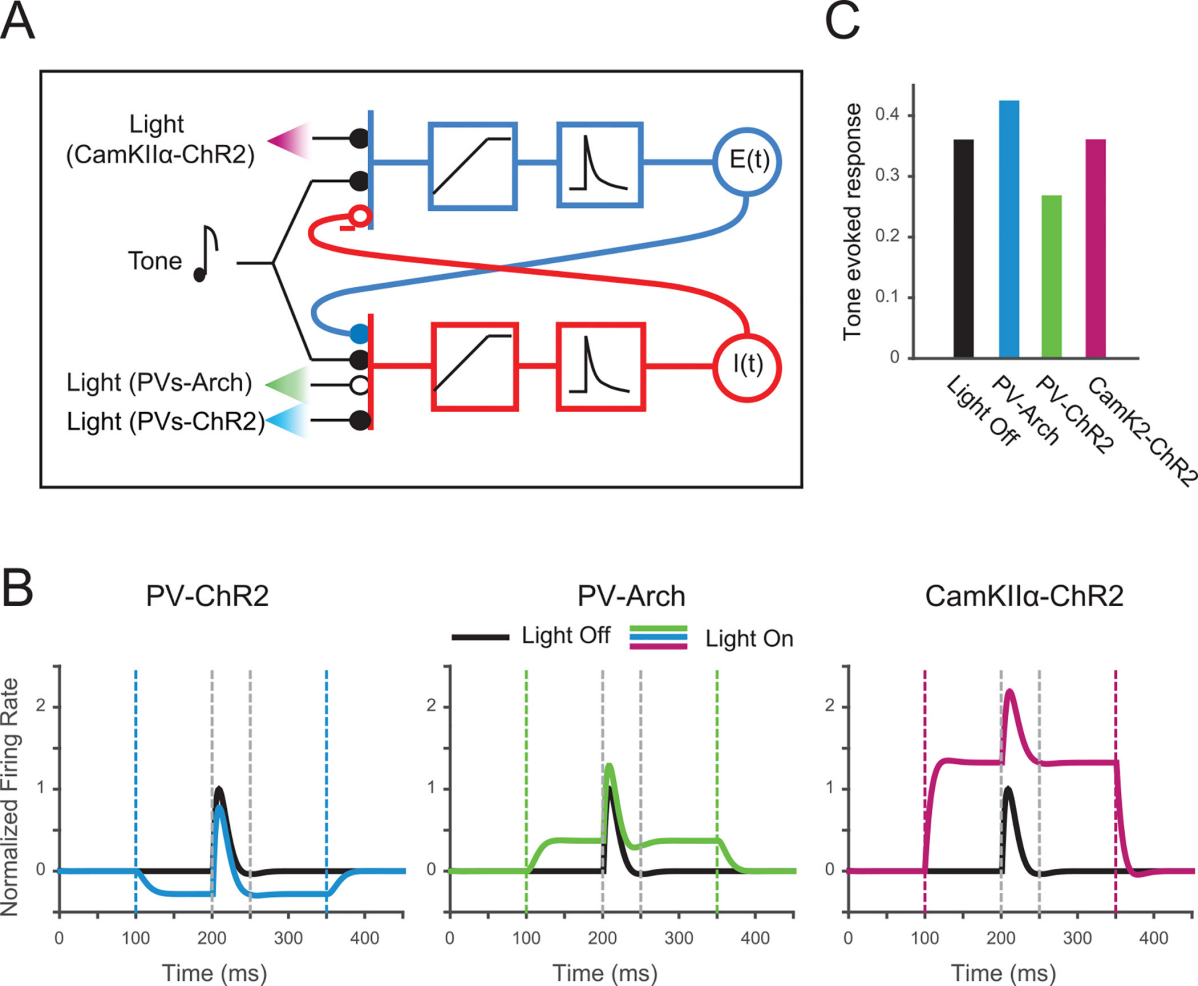
Mutually coupled excitatory–inhibitory neuronal model accounts for differential effects of PV and excitatory neuronal modulation on tone-evoked response magnitude. A. Diagram of model of inhibitory and excitatory mutually coupled neuronal populations. Closed circles: excitatory inputs; open circles: inhibitory inputs; -: depressing synapse. Blue boxes: excitatory pathway. Red boxes: inhibitory pathway. B. Tone-evoked responses of model neuronal excitatory population under different optogenetic manipulations. Tone is from 200 to 250 ms. Left: ChR2 in inhibitory neurons. Center: Arch in inhibitory neurons. Right: ChR2 in excitatory neurons. Black trace: Light-off condition; Color trace: Light-on condition. See matlab code in S1 Model. C. Mean magnitude of tone-evoked responses under different stimulation conditions.

We investigated a model of excitatory–inhibitory circuit interactions to better understand why manipulation of activity of PVs, but not excitatory neurons, affects the magnitude of tone-evoked responses. We constructed a firing-rate model as an extended Wilson-Cowan model of mutually connected excitatory and-inhibitory neuronal populations [53–55]. In this model, the inhibitory neuronal population integrates depolarizing currents from tone-evoked inputs and inputs from the excitatory neurons, whereas the excitatory neurons integrate tone-evoked inputs and hyperpolarizing currents from inhibitory neurons (Fig 6A, S14A Fig and Methods). Optogenetic modulation was modeled as an additional input current delivered to either excitatory or inhibitory neuronal populations. This simple simulation provided for a biological implementation of the circuit that is consistent with our experimental findings.

Inputs from PVs to excitatory neurons have been shown to exhibit synaptic depression [56,57]. We incorporated synaptic depression at the PV to excitatory synapse in the model (Fig 6). The model here did not assume a specific form (e.g., pre- or postsynaptic) of depression. Rather, we modeled synaptic transfer function as a nonlinearity, using a closed form solution for the relation between the output of the inhibitory neuronal firing rate and the input current for the excitatory neuronal population assuming depressing synaptic dynamics (see Methods). A simulation of excitatory neuronal responses exhibited the differential effects of inhibitory and excitatory stimulation of interneurons as well as lack of effect of stimulating the excitatory neurons directly on tone-evoked responses: Activating PVs increased the tone-evoked responses, whereas suppressing PVs decreased the tone-evoked responses of the excitatory population (Fig 6B and 6C). By contrast, activating excitatory population directly did not change the tone-evoked response magnitude (Fig 6B and 6C). This simulation thus provides for one plausible biological implementation of the circuit that is consistent with our experimental findings.

To develop a more basic understanding of the circuit, we implemented an instantaneous sigmoidal input–output nonlinearity with varying coefficients after synaptic integration for either excitatory or inhibitory neurons (S14 Fig). We used three different scenarios (S14A Fig): under scenario 1, the nonlinearity operates in a linear regime for both the excitatory and the inhibitory populations; under scenario 2, the nonlinearity is saturating for the excitatory, and linear for the inhibitory, neuronal population; under scenario 3, the nonlinearity operates in a saturating regime for the inhibitory population, and a linear regime for the excitatory population. Only scenario 3 (S14B–S14E Fig right) supported our experimental findings that a) suppressing PV activity increased the magnitude of tone-evoked responses (Fig 3A); b) increasing PV activity decreased the magnitude of tone-evoked responses (Fig 3C); and c) activating excitatory neurons directly did not affect tone-evoked response magnitude (but increased both the spontaneous and the tone-evoked firing rate by the same amount) (Fig 3E). Under scenario 1, activation of excitatory neurons did not affect tone-evoked response amplitude, but neither did activation or suppression of inhibitory neurons (S14B–S14E Fig, left). Under scenario 2, activation or suppression of inhibitory neurons decreased or increased tone-evoked response magnitude, respectively (S14B–S14E Fig center); however, activation of excitatory neurons decreased tone-evoked response magnitude. Therefore, the scenario 3, under which the excitatory neurons integrate their inputs close to linear, but the inhibitory inputs are passed through a saturating nonlinearity, is consistent with our data (S14B–S14E Fig right). The synaptic depression model (Fig 6) can be viewed as a special case of scenario 3, in which the transfer function between inhibitory and excitatory neuronal population saturates. Indeed, a number of other circuits, for example activation of an additional class of interneurons, such as somatostatin-positive interneurons [51], could potentially provide for a saturating transfer function.

## Discussion

Our results demonstrate that auditory cortical neurons regulate auditory behaviors that rely on frequency discrimination, and that this regulation can be facilitated by the overall activity level of a specific type of inhibitory, but not excitatory neurons. Optogenetic modulation of the level of activity of PV-positive interneurons drove changes in frequency discrimination acuity and specificity of auditory conditioning (Fig 2 and Fig 5). At the neuronal level, we find that modulating the level of PV activity differentially affects the spontaneous and the tone-evoked responses of putative excitatory neurons (Fig 1 and Fig 3). The changes in tone-evoked responses magnitude were correlated with behavioral performance (Fig 3G and Fig 5F). Whereas activating PVs during fear conditioning preserved specificity of conditioned fear, consistent with a previous pharmacological study [58], suppressing PVs increased generalization of fear responses. These effects of PVs extend beyond controlling the overall firing rate of excitatory neurons as changing the gain of excitatory neuronal responses directly did not lead to similar changes in behavioral performance (Fig 2G and Fig 5C). This difference can be attributed to a nonlinear relationship between inhibitory input from PVs and output firing rate of excitatory neurons, consistent with a mechanism of synaptic depression that has been identified at the synapse from PVs to excitatory neurons (Fig 6) [57]. Combined, our results support the view that PVs regulate signal-to-noise ratio of responses of principal neurons, extending beyond the effect of a global gain control, and that this dual effect on the spontaneous and tone-evoked activity affects behavioral frequency discrimination.

Our electrophysiological results demonstrating that activating PVs leads to narrower frequency tuning of putative excitatory neurons whereas suppressing PVs leads to broader frequency tuning (Figs 3 and 4) are consistent with previous pharmacological and electrophysiological investigations of inhibitory neuronal responses [59–63]. Behaviorally, while PVs have been implicated in two separate auditory behaviors: detection of temporal gap in sound [11] and in disinhibition of responses to tones during aversive stimulus presentation in AFC [43], our results provide for the initial demonstration of the role of PVs in auditory tasks relying on frequency discrimination. Our findings are thus consistent with those in the visual system, where PVs have been found to modulate responses of principal cells to visual stimuli and affect visual discriminative behavior [64–67].

Optogenetic approaches act on different timescales than lesion studies, or pharmacological methods for neuronal activity suppression. Lesioning or pharmacologically inactivating AC previously provided mixed effects on frequency discrimination, with some studies resulting in small, if any impairments in frequency discrimination performance [3,5,17,68], whereas other studies exhibited stronger effects [69]. These results are not inconsistent with the present findings: lesioning and pharmacological studies are performed on much longer time scales (hours to days [59]), as compared to the millisecond timescale of optogenetic perturbation. Therefore, lesioning or pharmacologically suppressing AC potentially allows for other neuronal circuits to take over frequency discrimination function, similarly to brain reorganization in response to injury [70] or simply abnormal lack of activity. Our results therefore support a modulatory, but not necessary, role for AC in frequency discrimination: when AC is “online”, excitatory–inhibitory circuits control frequency discrimination behavior, and their perturbation modulates frequency discrimination behaviors. By contrast, lesioning or suppressing AC pharmacologically for extended periods of time potentially allows for other brain areas to take over control of frequency discrimination.

Behavioral frequency discrimination acuity was tested through a task that is based on an innate, rather than learned response [3,40,71]. Implementing the PPI-based behavioral task has the advantage that the animal does not need to be trained on the task, and therefore allows for dissociation of perceptual report and learning. A recent study has found that corticocollicular feedback affects learning-induced changes in auditory spatial learning [72]. Here, similarly, AC may affect PPI through corticocollicular feedback, as PPI is controlled by the inferior-colliculus to pedunculopontine nucleus connection [39,73]. Future studies, including a test of the effect of inactivation of corticocollicular feedback, are needed to determine which of the possible circuits downstream of AC drive the observed behavioral changes.

Regulation of auditory frequency discrimination by the AC is not restricted to the PPI circuit, as we find that AC also regulates how specific conditioning is to a particular frequency of the tone. A number of studies have demonstrated that the AC plays an important role in fear conditioning [30]. Our results identify that the AC shapes frequency specificity of DAFC: suppressing the activity of interneurons decreased the specificity of DAFC, as the subjects generalized the conditioned response to the full range of tones on which they were tested (Fig 5). Several circuits may underlie this effect: AC projects to the amygdala, a crucial brain area in auditory fear conditioning, via the secondary AC or via feedback through the thalamus [41,74]. Applying selective manipulation to elements in these circuits in future studies will be necessary to learn how AC controls associative learning. Interestingly, activating PVs did not increase the specificity of auditory associative learning, measured by LS, as would have been expected from frequency discrimination results. This suggests that the limits to specificity of auditory associative learning may not only be set by the AC, but may also rely on other brain regions, which would have a lower frequency resolution than the AC. Furthermore, it points to an asymmetry between the effects of activation or suppression of circuit elements: taking out a crucial element of a circuit led to a qualitatively different effect than increasing the activity of an already present element.

Our results point to remarkable robustness of frequency discrimination to the overall level of activity in the AC. Whereas direct photoactivation of excitatory neurons dramatically increased the overall firing rate in the cortex, at the behavioral level, we did not observe a change in either behavioral frequency discrimination, as measured by *Th*, or in specificity of DAFC (Fig 2F and 2G, Fig 5C). This robustness to the mean firing rate level may underlie important perceptual effects, such as the ability to preserve acoustic discrimination or speech comprehension in different acoustic environments.

Our results provide for a mechanism by which the AC may modulate learning-driven changes in frequency discrimination following emotional learning [3]. Previously, we found that frequency discrimination acuity and specificity of learning were correlated across subjects, pointing to a common mechanism that controls the two behaviors. We identified AC as a candidate brain area for controlling frequency discrimination acuity and DAFC, as pharmacological inactivation of AC abolished DAFC-induced change in frequency discrimination acuity [3]. Inhibitory neurons differentially process auditory information and are affected by auditory learning and experience [13,75,76]. Our present results are consistent with the possibility that the learning-driven changes in frequency discrimination may be due to inhibitory interneuron activity or plasticity in inhibitory–excitatory connections.

Combined, we find that modulating frequency response properties of neurons in AC via activity of PVs modulates frequency discrimination acuity and specificity of auditory associative learning, confirming an important role for inhibitory circuits in AC in auditory behavior. While PVs are the most common type of interneurons in AC, other interneuron types, such as somatostatin-positive and vasoactive intestinal peptide-expressing inhibitory interneurons, may play additional complementary roles in shaping frequency discrimination, through more complex circuits. It will be important to tease apart the function of different cortical circuits in the processing of spectral information.

## Methods

### Animals

All experiments were performed in adult male mice (supplier: Jackson Laboratories; age, 12–15 wk; weight, 22–32 g; PV-Cre mice, strain: B6; 129P2-Pvalbtm1(cre)Arbr/J; CamKIIα-Cre: B6.Cg-Tg(CamKIIα-Cre)T29-1Stl/J; wild-type control: C57BL/6J) housed at 28°C on a 12 h light–dark cycle with water and food provided ad libitum, less than five animals per cage. In PV-Cre mice Cre recombinase (Cre) was expressed in PPI, and in CamKIIα-Cre, Cre was expressed in excitatory neurons [77]. All animal work was conducted according to the guidelines of University of Pennsylvanian IACUC and the AALAC Guide on Animal Research. Anesthesia by isofluorane and euthanasia by carbon dioxide were used. All means were taken to minimize the pain or discomfort of the animals during and following the experiments. All behavioral experiments were performed during the animals' dark cycle.

### Surgery and Virus Injection

At least 10 d prior to the start of experiments, mice were anesthetized with isoflurane to a surgical plane. The head was secured in a stereotactic holder. The mouse was subjected to a small craniotomy (2 x 2 mm) over AC under aseptic conditions. Viral construct was injected using syringe pump (Pump 11 Elite, Harvard Apparatus) targeted to AC (coordinates relative to bregma: −2.6 mm anterior, ±4.2 mm lateral, +1 mm ventral). Fiber-optic cannulas (Thorlabs, Ø200 μm Core, 0.22 NA) were implanted bilaterally over the injection site at depth of 0.5 mm from the scull surface. Craniotomies were covered with a removable silicon plug. A small headpost was secured to the skull with dental cement (C&B Metabond) and acrylic (Lang Dental). For postoperative analgesia, Buprenex (0.1 mg/kg) was injected intraperitonially and lidocaine was applied topically to the surgical site. An antibiotic (0.3% Gentamicin sulfate) was applied daily (for 4 d) to the surgical site during recovery. Virus spread was confirmed postmortem by visualization of the fluorescent protein expression in fixed brain tissue, and its colocalization with PV or excitatory neurons, following immuno-histochemical processing with the appropriate antibody.

### Viral Vectors

Modified AAV vectors were obtained from Penn VectorCore. Vector encoding light-gated proton pump Archaerhodopsin (Arch) under FLEX promoter was used for selective suppression of PVs (Addgene plasmid 22222, AAV-FLEX-Arch-GFP [35]). Modified AAV encoding ChR2 under FLEX promoter (Addgene plasmid 18917 AAV-FLEX-ChR2- tdTomato, ChR2 [78]) was used for activation of either PVs iin PV-Cre mice and or excitatory neurons in CamKIIα-Cre mice. Modified AAV vectors encoding only GFP or tdTomato under FLEX promoter were used as a control for the specific action of Arch and ChR2 on the neuronal populations.

### Histology

Brains were extracted following perfusion in 0.01 M phosphate buffer pH 7.4 (PBS) and 4% paraformaldehyde (PFA), postfixed in PFA overnight and cryoprotected in 30% sucrose. Free- floating coronal sections (40 μm) were cut using a cryostat (Leica CM1860). Sections were washed in PBS containing 0.1% Triton X-100 (PBST; 3 washes, 5 min), incubated at room temperature in blocking solution (10% normal goat serum and 5% bovine serum albumin in PBST; 3h), and then incubated in primary antibody diluted in blocking solution overnight at 4°C. The following primary antibodies were used: anti-PV (PV 25 rabbit polyclonal, 1:500, Swant), or anti-CAMKIIα (abcam5683 rabbit polyclonal, 1:500, abcam). The following day sections were washed in blocking solution (3 washes, 5 min), incubated for 1hr at room temperature with secondary antibodies (Alexa 594 or Alexa 488 goat anti-rabbit IgG; 1:1,000), and then washed in PBST (4 washes, 10 min). Sections were mounted using fluoromout-G (Southern Biotech) and confocal or fluorescent images were acquired (Leica SP5 or Olympus BX43). To quantify viral expression efficiency and specificity, cells in the proximity of injection site were identified in independent fluorescent channels and subsequently scored for colocalization using ImageJ’s cell counter plug-in.

### Photostimulation of Neuronal Activity

Neurons were stimulated by application of continuous light pulse delivered from either blue (473 nm, BL473T3-150, used for ChR2 stimulation) or green DPSS laser (532 nm, GL532T3-300, Slocs lasers, used for Arch stimulation) through implanted cannulas. Timing of the light pulse was controlled with microsecond precision via a custom control shutter system, synchronized to the acoustic stimulus delivery. Prior to the start of the experiment, the intensity of blue laser was adjusted to one of three values 0.2, 0.5, or 10 mW/mm^2^ as measured at the tip of the optic fiber. On average, the lowest light power was sufficient to induce significant reduction in *Th* (paired *t* test, t_19_ = 2.68, *p* = 0.015). However, in a small fraction of mice (6 out of 20), higher power was needed to induce reduction in *Th* (0.5 mW/mm^2^ in 5 mice, and 10 mW/mm^2^ in 1 mouse). The same power was used in auditory discriminative fear conditioning for each subject. Green laser was used at intensity of 10 mW/mm^2^, which resulted in similar absolute magnitude of change in spontaneous firing rate over the neuronal population as the lowest level of ChR2 activation (S3 Fig). An additional experiment carried out using 6 subjects (2 PV-Arch, 2 PV-ChR2 and 2 CAMKIIα-ChR2) demonstrated that photoactivation and suppression of neurons was confined to the AC (S2 Fig). The effect of light on firing rate significantly decayed over distance, but was heterogeneous over cortical depth (S2 Fig).

### Experimental Setup

During FC, the mouse was placed in a conditioning cage with a shock floor (Coulbourn) inside sound attenuation cubicle (Med Associates), housed in a single-walled acoustic chamber (Industrial acoustics). Throughout conditioning, the cage was illuminated with LED light, the color of which corresponded to the color of laser used for photoactivation of neurons (blue LED: 470 nm, 170 mW; green LED: 525 nm, 7 mW). During LS tests, a custom-made test cage of similar size but different floor and wall pattern and color was used. Auditory stimuli were provided by a free-field magnetic speaker (Tucker-Davis Technologies). Electric shock (0.5mA, 0.5 s) was delivered by precision animal shocker (Coulbourn). Freezeframe-3 software (Coulbourn) was used for stimulus control and analysis of freezing behavior.

During the PPI procedure, the mouse was placed in a custom-made tube on the sensor plate (San Diego Instruments) and head-fixed using implanted headpost. The speaker, housing, platform and webcam (Logitech) were placed in the sound attenuation cubicle (Med Associates), housed in a single-walled acoustic chamber. During tests, the housing was illuminated with LED light, the color of which corresponded to the color of the laser used for photoactivation of neurons. The speaker was positioned above the mouse. The sound delivery apparatus was calibrated using a 1/8-inch condenser microphone (Brüel&Kjær, Denmark) positioned at the expected location of the mouse's ear, to deliver each stimulus at 70 dB sound pressure level relative to 20 microPa (SPL). All pure tones presented during training and test sessions were at 70 dB SPL.

### Experimental Timeline

Seven to ten days after surgery, mice were subjected to at least three consequent days of habituation to experimental setups. During habituation to PPI apparatus, the duration of which gradually increased from 10 to 20 min over 3 d, mice were head fixed and optic fibers connected to cannulas. Following habituation, mice underwent daily PPI testing for frequency discrimination, which lasted for 1–3 d. Following PPI testing, a subset of mice underwent fear conditioning (FC) and one day thereafter they were tested for specificity of conditioned fear response. After termination of behavioral experiments, mice were used for electrophysiological recordings. In order to examine whether fear conditioning alters the effect of photoactivation on base firing rate of neurons and their tuning properties, we performed recordings in subgroup of PV-ChR2 mice without subjecting them to fear conditioning (“naïve” group, *n* = 6). Comparison of the change in spontaneous and tone-evoked firing rate and sparseness induced by photoactivation between “naïve” group and group that underwent fear conditioning did not reveal significant difference (S11 Fig). Therefore, recording data collected from these groups were pooled. All behavioral experiments were performed during animals’ dark cycle.

### Frequency Discrimination Acuity Test

The measurement of frequency discrimination acuity used a modified PPI of the startle reflex protocol as previously described [3,40]. The test measured the magnitude of the ASR to the startle stimulus (SS) as a function of the difference in frequency between the background tone and the prepulse tone (PP), which immediately preceded SS. The frequency of the background tone was 15.0 kHz. The background tone (when used) was presented continuously between the end of SS and the start of PP. The transition between the background tone and PP included 1 ms ramp to avoid clicks. Five frequencies used for PP (10.2, 12.6, 13.8, 14.7, and 15.0 kHz) were presented pseudo randomly with 10–20 s ISI, which also varied randomly. Thus, PP differed from the background tone by 0, 2, 4, 8, 16 and 32%. PP was 80 ms long and was presented right before SS. SS was broadband noise, presented at 100 dB SPL for 20 ms.

The magnitude of ASR was measured using a forcesensor plate (San Diego Instruments) and defined as the maximum vertical force applied within the 500 ms window following SS minus average baseline activity during 500 ms prior to SS. In each PPI session, 50% of the strongest ASRs for each frequency were averaged and used to calculate PPI:

$$PPI\left(\%\right)=100\frac{ASR_{noPP}-ASR_{PP}}{ASR_{noPP}}$$

where ASR_noPP_ is the response when PP frequency is equal to the frequency of the background tone (15 kHz) and ASR_PP_ is the response after frequency shift has occurred.

(a) To assess baseline frequency discrimination mice were subjected to the PPI procedure without photostimulation of neurons. Each test session consisted of 9 startle-only trials, followed by at least 100 pre-pulse trials, followed by one additional startle-only trial. On startle-only trials, background tone was followed directly by SS. On pre-pulse trials, each PP was presented 20 times in quasi-random order with ITI varying randomly between 10 and 20 s. Negative frequency changes were used because mice have been previously shown to be more sensitive to downward frequency shifts[3,40].

(b) To compare the effect of photoactivation or suppression of PVs on frequency discrimination, mice were subjected to a protocol similar to that described above, but including light delivery though implanted cannulas. On light ‘On’ trials, the laser was presented for 1 s, starting 0.5 s before PP onset. On light ‘Off’ trials laser was presented at quasi-random position during ITI. ‘On’ and ‘Off’ trials were shuffled randomly.

(c) To compare the subjective detectability of 5 experimental tones the background tone was omitted. The session started with 5 startle-only (no PP presentation) trials, followed by 50 pre-pulse trials, and terminated by 5 additional startle-only trials. On pre-pulse trials, each PP was presented 10 times in quasi-random order with ITI varying randomly between 10 and 20 s. The amplitude of each tone was then adjusted so that PPI induced by each tone was similar (S12 Fig).

The *Th* was defined as a frequency shift that caused 50% inhibition of the maximum ASR. *Th* is determined from a parametric fit to a generalized logistic function:

$$PPI=\frac{a}{1\;+\text{exp}\left(b+c\Delta f\right)}$$

In a standard PPI session, 20 repetitions of each PP were presented (100 trials in total). However, if either *Th* was out of the range (0.5–32%) or the fit coefficient of the curve (R^2^) was below 0.7, the mouse underwent an additional 10 repetitions (50 trials). If *Th* and fit curve failed to meet the above criteria after 200 trials, the session was excluded from statistical analysis (3 out of 61 sessions).

### Fear Conditioning

During FC, following 5 min of silence, 10 tones (15.0 kHz, 10.5 s) co-terminated with a foot shock (CS+) were presented, at an inter-trial interval (ITI) randomly varied between 2 to 6 min. In addition, 10 tones at 11.25 kHz (10.5 s), not paired with foot-shock (CS-) were presented in random order with 2 min inter-stimulus interval (ISI). Photoactivation and suppression of neurons was performed by delivery of light through implanted cannulas. In one group of mice, photo stimulation started 0.5 s before CS+ and CS- onset and co-terminated with the tone (11 s total). In another group, photo stimulation was terminated 1 s before the tone offset to avoid overlapping with the foot-shock (10 s total).

### LS Test

The LS test consisted of CS+ and three test tones (3.75, 7.5, 11.25 kHz), presented 3 times at 3 min ISI. LS was assayed as the difference in freezing response to CS+ and mean freezing response to three test tones:

$$LS\left(\%\right)=100\frac{F_{CS+}-\langle F_{test}\rangle }{F_{CS+}}$$

Where F_CS+_ is freezing (%) during CS+ tone presentation and F_test_ is mean freezing during test tones.

During conditioning and test sessions, freezing responses were video-recorded and analyzed offline using Freeze Frame software. Freezing responses were judged as complete immobility of the mouse for at least 1 s. Average freezing response during 20 s before the test tones was recorded as baseline, while freezing response during the test tones was recorded as the conditioned response. Subjects that exhibited either very low conditioned freezing to CS+ tone (<20%, n = 2) or very low locomotion throughout the test (>50%, n = 1) were excluded from statistical analysis.

During conditioning, photostimulation was presented during CS+ and, in most subjects, terminated 0.5 s before the onset of the footshock. However, in a subset of mice (ChR2: N = 5, Arch: N = 4), photostimulation overlapped with the footshock (S13A Fig). While overlapping the photostimulation with the footshock affected the freezing response in PV-ChR2 group (S13B Fig), as previously described [43]), it did not result in a significant difference in LS (S13C Fig). In PV-Arch group, we did not observe significant effect of the overlap of photostimulation with the footshock on either freezing response (S13D Fig) or LS (S13E Fig). Therefore, the two subsets of mice were combined for subsequent analysis within each group.

### Electrophysiological Recordings

All recordings were carried out inside a double-walled acoustic isolation booth (Industrial Acoustics). Mice were placed in the recording chamber, and a headpost was secured to a custom base, immobilizing the head. Activity of neurons in the primary AC was recorded via a silicon multi-channel probe (Neuronexus), lowered in the area targeting AC via a stereotactic instrument following a durotomy. The electrode tips were arranged in a vertical fashion that permits recording the activity of neurons in different cortical laminae. Electro-physiological data from 32 channels were filtered between 600 and 6000 Hz (spike responses), digitized at 32kHz and stored for offline analysis (Neuralynx). Spikes belonging to single neurons were detected using commercial software (Plexon) [79].

### Acoustic Stimulus

Stimulus was delivered via a magnetic speaker (Tucker-David Technologies), calibrated with a Bruel and Kjaer microphone at the point of the subject's ear, to deliver tones at frequencies between 1 and 80 kHz to +- 3 dB [79]. To measure the frequency tuning curves, we presented a train of 50 pure tones of frequencies spaced logarithmically between 1 and 80 kHz, at 8 intensities spaced uniformly between 10 and 80 dB, each tone repeated twice in pseudo-random sequence, counter-balanced for laser presentation. The full stimulus was repeated 5 times. Each tone was 50 ms long, with inter-stimulus interval (ISI) of 450 ms. The light-Onset was presented during every other tone, with the onset of 100 ms prior to tone onset, and lasting for 250 ms.

### Neuronal Response Analysis

The effect of the light-On FR was assessed by as an index of change in FR in light-On and light-Off trials:

$$\Delta FR=\frac{FR_{ON}-FR_{OFF}}{FR_{ON}+\;FR_{OFF}}$$

The change was computed separately for the spontaneous and tone-evoked firing rate. The spontaneous firing rate (FR_base_) was computed by averaging FR over 50 ms before tone-Onset across light-On and light-Off trials. The tone-evoked firing rate (FR_tone_) was computed as the average of FR of responses to tones at 60–80 dB SPL at 0–50 ms after tone onset were averaged. To examine frequency selectivity of neurons, sparseness of frequency tuning was computed as:

$$Sparseness=1-\frac{{\left(\sum_{i=1}^{i=n}\;FR_{i}/n\right)}^{2}}{\sum_{i=1}^{i=n}\;FR_{i}{}^{2}/n}$$

where *FR*_*i*_ is tone-evoked response to tone at frequency *i*, and *n* is number of frequencies used.

To compute the width and BF of tuning, the frequency response function was fitted Gaussian function:

$$FR\left(f\right)=a\;e^{-\frac{{\left(f-f_{b}\right)}^{2}}{2\sigma^{2}}}$$

where *f*_*b*_ is the BF and *σ* is the standard deviation of the Gaussian function. The tuning width was measured in octaves as the difference between *f*_*b*_ + *σ* and *f*_*b*_ − *σ* for neurons, for which the Gaussian fit had *R* ^*2*^>0.4.

Magnitude of neuronal response to tones was defined as the difference between mean spontaneous (0–50 ms before tone onset) and tone-evoked (0–50 ms after tone onset) firing rate and, for each neuron, normalized by setting the peak response magnitude between 0 and 50 ms after tone onset on light-Off trials to 1. Only responses to tones within 0.5 octaves of BF of each neuron were included. To quantify correlation between neuronal responses and behavioral frequency discrimination, normalized tone-evoked response magnitude over all neurons recorded in each mouse was compared to changes in behavioral *Th*. Only mice with >5 identified single units (33 out of 36 mice) were used for statistical analysis.

### Identification of Putative Excitatory Neurons

First, we determined the criteria based on the spike waveform analysis. Putative PV interneurons in PV-ChR2 mice were preselected for waveform analysis if they exhibited a significant (more than 2-fold) increase in firing rate in response blue light (10 mW/mm^2^) and their spontaneous firing rate exceeded 3 Hz. Waveform analysis showed that spikes of these neurons have relatively low peak to trough amplitude ratio (<1.2, S1D Fig) consistently with previous reports [50]. In order to exclude PV interneurons from the pool of neurons used for the analysis of tone-evoked responses, only neurons with peak to trough ratio that exceeded 1.2 were used. In addition, putative excitatory cells were identified based on their expected response patterns to sounds and lack of significant activation of the spontaneous firing rate by the laser in PV-ChR2 mice and suppression in PV-Arch mice [50,80]. While this subpopulation may still contain inhibitory neurons, the proportion of interneurons recorded was relatively small, as we used silicon electrode probes with relatively low impedance that do not target interneurons [50]. The low impedance of the probes precluded us from conducting a more detailed analysis for fast-spiking versus regular-spiking neurons based on the spike waveform [50].

### Excitatory–Inhibitory Network Model

We constructed a model of the excitatory-inhibitory neuronal circuit based on a firing rate model, based on Wilson-Cowan dynamics [53–55]. The mean activity level of each population was modeled as:

$$\frac{\text{dE}}{\text{dt}}=\frac{1}{\tau_{\;\text{E}}}\left[-\text{E}\left(\text{t}\right)+\left(\text{k}-\text{r}\right)\text{S}\left({\text{j}}_{\text{CamK}2}\left(\text{t}\right)+{\text{j}}_{\text{ETone}}\left(\text{t}\right)+{\text{S}}_{inh}({\text{j}}_{\text{IE}}\text{I}\left(\text{t}\right)\right)\right]$$

$$\frac{\text{dI}}{\text{dt}}=\frac{1}{\tau_{\;\text{I}}}\left[-\text{I}\left(\text{t}\right)+\left(\text{k}-\text{r}\right)\text{S}\left({\text{j}}_{\text{PV}}\left(\text{t}\right)+{\text{j}}_{\text{ITone}}\left(\text{t}\right)+{\text{j}}_{\text{EI}}\text{E}\left(\text{t}\right)\right)\right]$$

where E(t) is the firing rate of the excitatory population; I(t) is the firing rate of the inhibitory population; S(x) is the firing transfer function between the combined postsynaptic input and the neuronal firing rate; S_*inh*_(*x*) is the transfer function between the inhibitory firing rate and excitatory postsynaptic current; j_EI_ (0.2) and j_IE_ (−0.2) are excitatory–inhibitory and inhibitory–excitatory synaptic weights; j_ETone_(t) and j_ITone_(t) are tone-evoked input currents to excitatory and inhibitory neurons, respectively, modeled as 50 ms long exponentially decaying inputs of maximum amplitude 3; τ _E_ (10 ms) and τ _I_ (10 ms) are synaptic time constants for excitatory and inhibitory neurons; k and r represent the maximum and minimum firing rates of neurons respectively (k = 15, r = 1); j_CamK2_(t) is the input to excitatory neurons due to ChR2-driven activation; j_PV_(t) is the input to inhibitory neurons due to either ChR2 (positive) or Arch (negative). The optogenetic modulation was modeled as a unitary pulse of 250 ms in duration. We simulated activation of inhibitory neurons by setting j_PV_(t) = 1, activation of excitatory neurons j_CamK2_(t) = 1.5, or suppression of inhibitory neurons by setting j_PV_(t) = −1.

The transfer functions is given by:

$S(x)=\frac{\left(x-a\right)}{\left(b-a\right)}$ for *a* < *x* < *b*; *S*(*x*) = 0 for *x* < *a*; *S*(*x*) = 1 for *x* > *b*; where for excitatory neurons, a = −2, b = 1.5; for inhibitory neurons, a = 0, b = 4.

For the inhibitory-to-excitatory inputs, we used a simplified saturating transfer function, *S*_*inh*_(*x*), which is the quasistatic solution to a differential equation for the synaptic conductance *g* with depletion and replenishment given by:

$$\frac{dg}{dt}=-gr/T_{d}+(g_{0}-g)/T_{r}$$

Here, *r* is the presynaptic firing rate, *g* is the synaptic conductance, *g*_0_ is the maximum conductance, and *T*_*d*_ and *T*_*r*_ are the time constants for depletion and replenishment, respectively. The input to the post-synaptic neuron is given by the product *gr*. Then, $S_{inh}(x)=\frac{gx}{1+cx}$where g = 2, c = 0.15.

For visualization, the firing rate of neurons was normalized as in Fig 3A, 3C and 3E, by subtracting the baseline firing rate, and setting the peak of the tone-evoked firing rate to 1 on light-off trials.

### Statistical Analysis

Because most of behavioral experiments consisted of within-subject repeated measurements, most of the data were analyzed by either two-tailed paired *t* test or repeated-measures ANOVA using SPSS Statistics (IBM) or Matlab (Mathworks). The effect of photoactivation and inactivation of neuronal activity on tuning properties was examined using a one-sample *t* test. Samples that did not pass Shapiro-Wilk test for normality were compared using Wilcoxon signed rank test. Multiple comparisons were adjusted by Bonferroni correction. Equality of variances was confirmed using Levene’s test.

## Supporting Information

S1 Data Excel file containing data for the key figures in the article. (XLSX)

S1 Fig Analysis of light-evoked responses of putative PV interneurons. (A) Peristimulus time histograms (PSTH) of sample putative PV neurons activated (blue, PV-ChR2 mouse) or inhibited (green, PV-Arch) by 250-ms-long pulse of light (outlined by gray rectangle). Inset shows a raster plot of a putative PV interneuron activated by blue light with a short latency. Light is presented between 0 and 25 ms (blue rectangle). (B) Effect of photostimulation on spontaneous firing rate (FR_base_) of putative PV interneurons expressing ChR2 (blue) and Arch (green). Units shown in (A) are circled. (C) Scatter plot of the spike width at half height plotted against peak to trough amplitude ratio for putative PV+ (blue) and PV- (black) neurons. Insets show mean ± SEM. waveforms for PV+ (blue) and PV- (black) neurons. Units with FR_base_ higher than 3 Hz, whose photoactivation exceeded 200% were identified as PV+ neurons.
(TIF)

S2 Fig The effect of photostimulation on auditory responses as a function of distance from optocannula and depth. We measured the effect of light activation on multiunit activity during noise bursts. We recorded neuronal activity in the AC of head-fixed awake mice at 0, 0.4, 0.8 and 1.2 mm from the optocannula and at 6 depths between 0 and 1.25 mm from brain surface. The sound stimulus was a 50 ms long white noise burst. On half the trials, the sound was accompanied by a 250 ms long light pulse emitted from the optocannula, which started 100 ms prior to sound onset. Multiunit clusters were identified using Plexon online spike-sorter, and their firing rate was computed on light-off and light-on trials (S2A-SC Fig). We computed the percentage of units whose baseline firing rate (0–50 ms pre light onset) was increased (PV-Arch and CamKIIα-ChR2 groups) or decreased (PV-ChR2) due to light (0–50 ms post light onset) and the index of change of their mean firing rate during white-noise burst (0–50 ms post noise burst onset) on light-on as compared to on light-off trials (ΔFR_sound_). In all three groups, the effect of light on sound-evoked multiunit activity significantly declined over distance (S2D-S2F Fig one-way ANOVA with distance as factor, PV-ChR2: *F*_3,535_ = 52.28, *p* = 1.3e-29; PV-Arch: *F*_3,437_ = 3.34, *p* = 0.019; CamKIIα-ChR2: *F*_3,555_ = 4.26, *p* = 0.005). The effect of light was stronger in the CamKIIα-ChR2 than in PV-Arch group across all distances (two-way ANOVA: effect of group, *F*(df = 1) = 34.24, *p* < 0.0001; effect of distance, *F*(df = 3) = 4.58, *p* = 0.0038; interactions, group x distance, *p* > 0.05). The effect of the light on multiunit activity as a function of depth was heterogeneous (S2D-S2F Fig). In all three groups, one-way ANOVA with depth as factor was not significant. In PV-ChR2 and PV-Arch groups, change in FR exhibited an inverted U-shape dependency on the depth,declining significantly from 0.5–0.75 mm to 1.25 mm (two-sample *t* test, PV-ChR2: *t*_101_ = −5.3, *p* = 8.3e-7; PV-Arch: *t*_190_ = 2.1, *p* = 0.039). For CamKIIα-ChR2 mice, the dependency exhibited a more linear pattern with the effect of light declining significantly from 0–0.25 to 1.25 mm (two-sample *t* test, PV-ChR2: *t*_122_ = 2.2, *p* = 0.033). The effect of light was stronger in the CamKIIα-ChR2 than in PV-Arch group across all depths (two-way ANOVA: effect of group, *F*(df = 1) = 36.18, *p* < 0.0001; effect of depth, F(df = 5) = 1.6, *p* >0.05; interactions, group x depth, *p* > 0.05.). A, B, C. Average time course of normalized multiunit activity in response to stimulation by light (outlined by color dashed lines) and noise (outlined by black dashed lines) on light-On (color) and light-Off (gray) trials for sound-responsive units whose firing rate was elevated during sound presentation as compared to baseline. Top plots depict responses within the range of 0–0.4 mm from the cannula. Bottom plots depict responses within the range of 0.8–1.2 mm from cannula. Mean ± SEM. Data from multiunits normalized to 0 at baseline and 1 for peak firing rate (similar to normalization of single unit firing rates in Fig 3). A. PV-ChR2 mice (*n* = 2, blue). Top: 122 units. Bottom: 182 units. B. PV-Arch mice (*n* = 2, green). Top: 212 units. Bottom: 116 units. C. CamKIIα-ChR2 mice (*n* = 2, magenta). Top: 215 units. Bottom: 104 units. D, E, F. The effect of the laser on multiunit activity decreases as a function of horizontal distance from the optic fiber. Right: Fraction of units inhibited (PV-ChR2) or activated (PV-Arch and CamKIIα-ChR2) by light. Left: ΔFR_sound_ as a function of horizontal distance from optic fiber. Data were obtained by four penetrations of multi-electrode probes at distance of 0, 0.4, 0.8, and 1.2 mm from optic cannula. D. PV-ChR2 (*n* = 2 mice). E. PV-Arch (*n* = 2 mice). F. CamKIIα-ChR2 (2 mice). G, H, I. Heterogeneous effects of the effect of the laser on multiunit activity as a function of depth from the brain surface. Right: Fraction of units inhibited (PV-ChR2) or activated (PV-Arch and CamKIIα-ChR2) by light. Left: ΔFR_sound_ as a function of depth from the brain surface. Data were obtained by gradual lowering of a linear multielectrode probe twice, resulting in recordings in the range between 0 and 1.25 mm from the brain surface. G. PV-ChR2 mice (2 mice, 166 units). H. PV-Arch mice (2 mice, 303 units). I. CamKIIα-ChR2 mice (2 mice, 342 units).
(TIF)

S3 Fig Light intensity–dependent effect of PVs photostimulation on neuronal activity in PV-ChR2 mice. (A) Light intensity. Left: 0.2 mW/mm^2^ (*n* = 330 neurons); middle: 0.5 mW/mm^2^ (*n* = 322 neurons); right: 10 mW/mm^2^ (*n* = 202 neurons). Each circle represents a single unit. Spontaneous firing rate (FR_base_) was suppressed as result of photoactivation of PVs in a light-intensity-dependent fashion (FR_base_ on light-On is plotted versus light-Off trials). (B) Mean index of change in spontaneous firing rate due to different intensity of photoactivation of PVs over neuronal population. ***: One-way ANOVA, *F*_2,851_ = 156.38, *p* = 1.5e-58. (C) Tone-evoked firing rate (FR_tone_) is suppressed during photostimulation (light-On versus light-Off trials). Columns as in (A). (D) Mean index of change in FR_tone_ due to different intensity of photostimulation of PVs over neuronal population. ***: One-way ANOVA, *F*_2,851_ = 48.35, *p* = 1.3e-20.
(TIF)

S4 Fig Design of behavioral test for frequency discrimination acuity. The test relied on measurement of inhibition of auditory startle response by PPI. (A) Time course of acoustic stimulation during a single PPI trial. Three stimuli were presented in succession: 1) background tone at frequency (f1) identical to CS+ used in fear conditioning; 2) prepulse tone at the same amplitude but different frequency (f2) than the background tone; 3) startle broadband noise that evoked a startle response. (B) Parameters of stimuli used in PPI. Note that the duration of the background tone varied randomly between 10 and 20 s. On each trial, prepulse tone was presented at a frequency randomly selected from five listed frequencies. (C) Sample PPI versus Tone frequency shift curve. Reduction in the magnitude of the startle response (% PPI) increased as a function of frequency shift (%) between the background and prepulse tone. Each data point represents the average PPI over at least ten trials. Red dashed line is the logistic fit curve (see Methods). *Th* was defined as the frequency shift at 50% maximum PPI.
(TIF)

S5 Fig Photostimulation does not affect frequency discrimination acuity in mice expressing control viral constructs. (A) Behavioral frequency discrimination acuity as measure by *Th* is not affected by photostimulation with blue light in mice expressing control viral constructs (*n* = 6). Left. PPI as a function of frequency shift in light-On (blue) and light-Off (black) condition. Right. Mean *Th* values (blue) and *Th* for each subject (gray) in light-On and light-Off condition, and in the session where no photostimulation was presented (“No light”). (B) Behavioral *Th* is not affected by photostimulation with green light in mice expressing control viral constructs (*n* = 6). Left. PPI as a function of frequency shift in light-On (green) and light-Off (black) condition. Right. Mean *Th* values (green) and *Th* for each subject (gray) in light-On and light-Off condition, and in the session where no photosuppression was presented (“No light”). Axes: same as in Fig 2C and 2E. Blue light: paired *t* test, t_5_ = −0.55, *p* = 0.1. Green light: paired *t* test, *t*_5_ = −1.35, *p* = 0.24.
(TIF)

S6 Fig Neither activation (top) nor suppression (bottom) of PVs affected basic PPI parameters. (A) Startle response magnitude in the absence of prepulse signal (no frequency shift between background and prepulse tones) on light-On (color bars) and light-Off trials (gray bars) averaged across mice from PV-ChR2 and PV-Arch groups. ns: Difference not significant (paired *t* test. PV-ChR2: *n* = 20, *t*_19_ = 0.365, *p* = 0.719; PV-Arch: *n* = 16 *t*_15_ = −0.86, *p* = 0.41). (B) Maximum PPI values induced by prepulse frequency shift on light-On (color bars) and light-Off trials (gray bars) averaged across mice from PV-ChR2 and PV-Arch groups. ns: Difference not significant (PV-ChR2: paired *t* test, *t*_19_ = −0.63, *p* = 0.535; PV-Arch: paired *t* test, *t*_15_ = −1.9, *p* = 0.083). Each bar represents average across subjects ± SEM.
(TIF)

S7 Fig PPI elicited by tones of different frequencies in the absence of background tone. Neither activation (A) nor inhibition (B) of PV interneurons affected PPI induced by tones of different frequency without background tone. These results indicate that photostimulation did not change subjective loudness of tones in frequencies used in frequency discrimination test. Comparison of PPI for light-On (color bars) and light-Off trials (gray bars) revealed no significant difference in either group (two-way ANOVA-light effect, PV-ChR2: *n* = 598 trials, *F*_1,586_ = 0.52, *p* = 0.47; PV-Arch: *n* = 602 trials, *F*_1,590_ = 2.1, *p* = 0.14). Comparison of PPI elicited by prepulse tones of six different frequencies did not reveal significant difference (two-way ANOVA-frequency effect, PV-ChR2: *F*_5,586_ = 0.90, *p* = 0.47; PV-Arch: *F*_5,590_ = 1.7, *p* = 0.14). (A) Data for mice from PV-ChR2 group (*n* = 4 mice). (B) Data for mice from PV-Arch group (*n* = 5 mice). Each bar represents mean ± SEM across subjects.
(TIF)

S8 Fig Effect of direct photoactivation of excitatory neurons in CamKIIα-ChR2 mice on neuronal auditory responses. (A) Photoactivation of CamKIIα neurons leads to a significant increase in FR_base_ of putative excitatory neurons. Top: index of change in the FR_base_ across neuronal population. Bottom: FR_base_ in light-On trials versus light-Off trials. ***: one-sample *t* test, *t*_205_ = 11.84, *p* = 5.4e-25, mean ΔFR_base_ = 0.27. (B) Photostimulation increased the tone-evoked firing rate. Top: histogram of the index of change in the tone-evoked firing rate (FR_tone_) across neuronal population. Bottom: FR_tone_ in light-On trials plotted versus light-Off trials. ***: *t*_205_ = 6.71, *p* = 1.9e-10, mean ΔFR_tone_ = 0.11.
(TIF)

S9 Fig Light intensity–dependent effect of PVs photostimulation on sparseness of tuning in PV-ChR2 mice. (A) Light intensity. Left: 0.2 mW/mm^2^ (*n* = 215 neurons); middle: 0.5 mW/mm^2^ (*n* = 240 neurons); right: 10 mW/mm^2^ (*n* = 175 neurons). Each circle represents a single auditory unit suppressed by light. Sparseness of tuning increased due to photoactivation of PVs in a light intensity-dependent fashion (sparseness on light-On is plotted versus light-Off trials). (B) Mean index of change in sparseness during photostimulation of PVs at varying light intensities. ***: One-way ANOVA, F_2,851_ = 38.2, *p* = 1.3e-16. (C) Index of change in sparseness of neuronal responses resulting from different levels of laser stimulation as a function of change in FR_base_. 0.2 mW/mm^2^: *p* = 1.5e-23; 0.5 mW/mm^2^: *p* = 4.2e-21; 10 mW/mm^2^: *p* = 8.9e-12. Columns as in (A).
(TIF)

S10 Fig Diagram of the DAFC protocol. (A) A discriminative fear conditioning session consisted of 10 presentations of a 15 kHz tone (CS+) coterminated with a mild foot-shock (unconditioned stimulus, US). In addition, 10 unpaired tones (11.25 kHz, CS) were presented along with CS+ with 2 min interstimulus interval (ISI). Randomized inter-trial interval (ITI, time between CS+ presentations) was 2,4, or 6 min. (B) The LS test was carried out in a different context from conditioning. The LS test consisted of CS+ and three test tones (3.75, 7.5, 11.25 kHz), presented in random order three times each at 3 min ISI. LS was assayed as the differential freezing response to CS+ and test tones (Methods). (C) Peristimulus time histogram (PSTH) of putative PV- neurons in response to 10-s-long laser pulses (outlined by gray rectangle) in PV-Arch (green) and PV-ChR2 (blue) mice.
(TIF)

S11 Fig Undergoing DAFC does not alter the effect of PV activation on neuronal responses. (A) Diagram of experimental procedure for naïve animals (top) and animals that underwent DAFC prior to recording. Change in spontaneous and tone-evoked firing rate (B) and change in sparseness of neuronal tuning due to photoactivation of PVs (C) were similar for mice that underwent DAFC and naïve animals (MANOVA with conditioned or naive subject as a factor (F_1,628_ = 1.0, *p* = 0.49)). Gray dots: results of electrophysiological recording from each neuron in naive subjects (*n* = 6). Red dots: results from electrophysiological recording from each neuron 2–5 days after fear conditioning (*n* = 8).
(TIF)

S12 Fig Subjective tone loudness used for frequency discrimination is similar across all used frequencies. Perception of tone loudness was estimated as PPI elicited by prepulse tone without background tone. Each bar represents mean ± SEM across mice in PV-ChR2 group (A, *n* = 20, repeated-measures ANOVA, *p* = 0.066) and PV-Arch group (B, *n* = 16, *p* = 0.52).
(TIF)

S13 Fig Overlap between photoactivation of PVs with the US presentation during fear conditioning altered conditioned response (freezing) during test but did not affect how specific associative learning was as measured by LS. (A) In US+light group, blue (left) or green (right) light stimulation was 11 s long and overlapped with the presentation of electric foot-shock (US). In US-light group, light stimulation lasted for 10 s and terminated 0.5 s before the US onset. (B) In PV-ChR2 group, activation of PVs during US presentation (US+light) significantly reduced freezing during test session as compared to US-light group. *: *p* = 0.025, *t* test, *t*_14_ = 2.53,. (C) In PV-ChR2 group, activation of PVs during US presentation (US+light) did not affect specificity of conditioned response. ns: *t* test, *t*_14_ = 0.67, *p* = 0.51. (D and E) In PV-Arch group, inhibition of PVs during US presentation (US+light) did not significantly affect either freezing (d) (*t* test, *t*_11_ = 1.16, *p* = 0.27) or LS (e) (*t* test, *t*_11_ = 0.45, *p* = 0.66).
(TIF)

S14 Fig Coupled excitatory–inhibitory neuronal model requires a saturating synaptic transfer function for inhibitory population to replicate experimental findings. (A) Three additional models that were used to model the excitatory-inhibitory coupled networks. Left: the synaptic transfer function was modeled as linear for both excitatory and inhibitory population. Center: The synaptic transfer function was modeled as saturating for excitatory, and linear for inhibitory population. Right: The synaptic transfer function was modeled as saturating for inhibitory and linear for excitatory population. (B–D) Responses of excitatory neuronal population to a 50-ms long tone presented at 200 ms with (color) and without (black) optogenetic stimulation (color, btw. 100–350 ms). B. Blue: activation of inhibitory neurons. C. Green: suppression of inhibitory neurons. D. Magenta: Activation of excitatory neurons. Compare to Fig 3A, 3C and 3E. (E) Mean tone-evoked magnitude (mean firing rate during tone—spontaneous firing rate just preceding the tone). Colors as in B–D. Compare to Fig 3B, 3D and 3F.
(TIF)

S1 Model Matlab code for model presented in Fig 6 and S14 Fig. (ZIP)

## Abbreviations

AAV: adeno-associated virus
AC: auditory cortex
Arch: archaerhodopsin
ASR: acoustic startle response
BF: best frequency
ChR2: channelrhodopsin
DAFC: differential auditory fear conditioning
LS: learning specificity
PPI: prepulse inhibition
PV: parvalbumin-positive interneurons
SEM: standard error of the mean
*Th*: frequency discrimination threshold
