# Supplementary figures and images for: Bidirectional Regulation of Innate and Learned Behaviors That Rely on Frequency Discrimination by Cortical Inhibitory Neurons
Source: PLoS Biol. 2015 Dec 2;13(12):e1002308. doi: 10.1371/journal.pbio.1002308 (PMC4668086; doi:10.1371/journal.pbio.1002308)

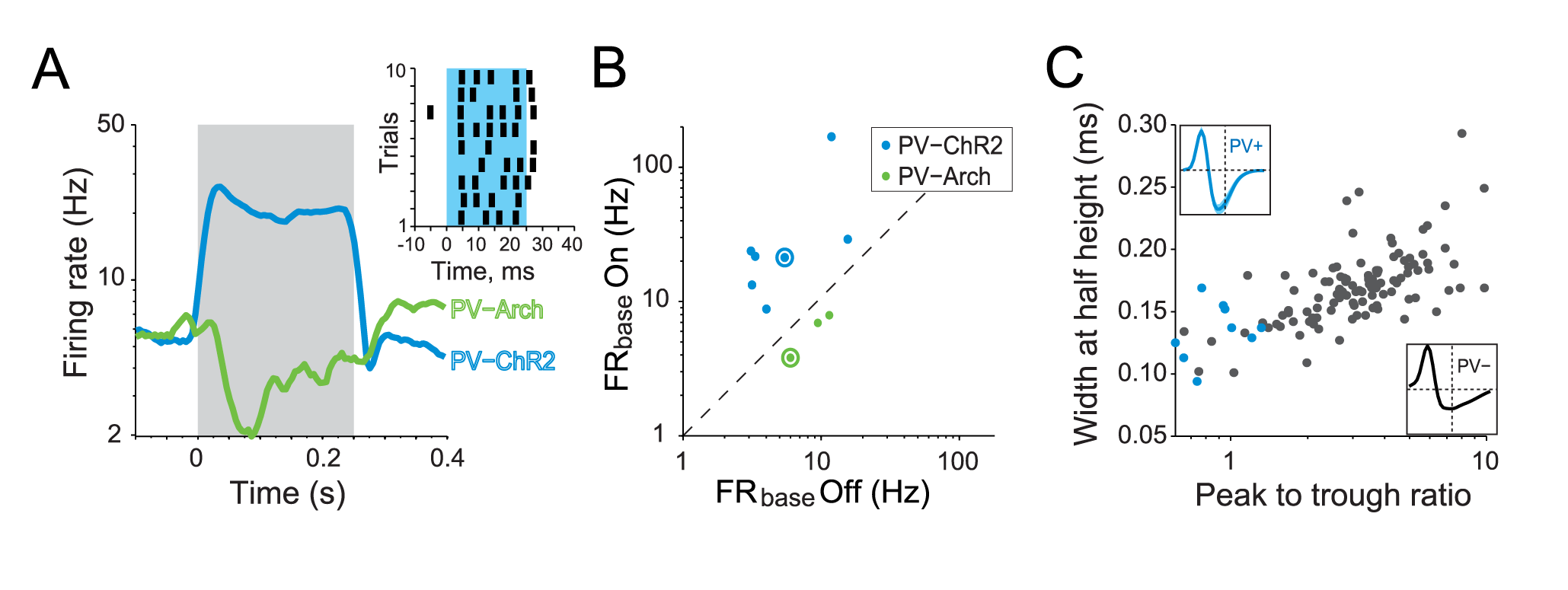

Supplement: S1 Fig — (A) Peristimulus time histograms (PSTH) of sample putative PV neurons activated (blue, PV-ChR2 mouse) or inhibited (green, PV-Arch) by 250-ms-long pulse of light (outlined by gray rectangle). Inset shows a raster plot of a putative PV interneuron activated by blue light with a short latency. Light is presented between 0 and 25 ms (blue rectangle). (B) Effect of photostimulation on spontaneous firing rate (FRbase) of putative PV interneurons expressing ChR2 (blue) and Arch (green). Units shown in (A) are circled. (C) Scatter plot of the spike width at half height plotted against peak to trough amplitude ratio for putative PV+ (blue) and PV- (black) neurons. Insets show mean ± SEM. waveforms for PV+ (blue) and PV- (black) neurons. Units with FRbase higher than 3 Hz, whose photoactivation exceeded 200% were identified as PV+ neurons. (TIF) [file pbio.1002308.s002.tif]

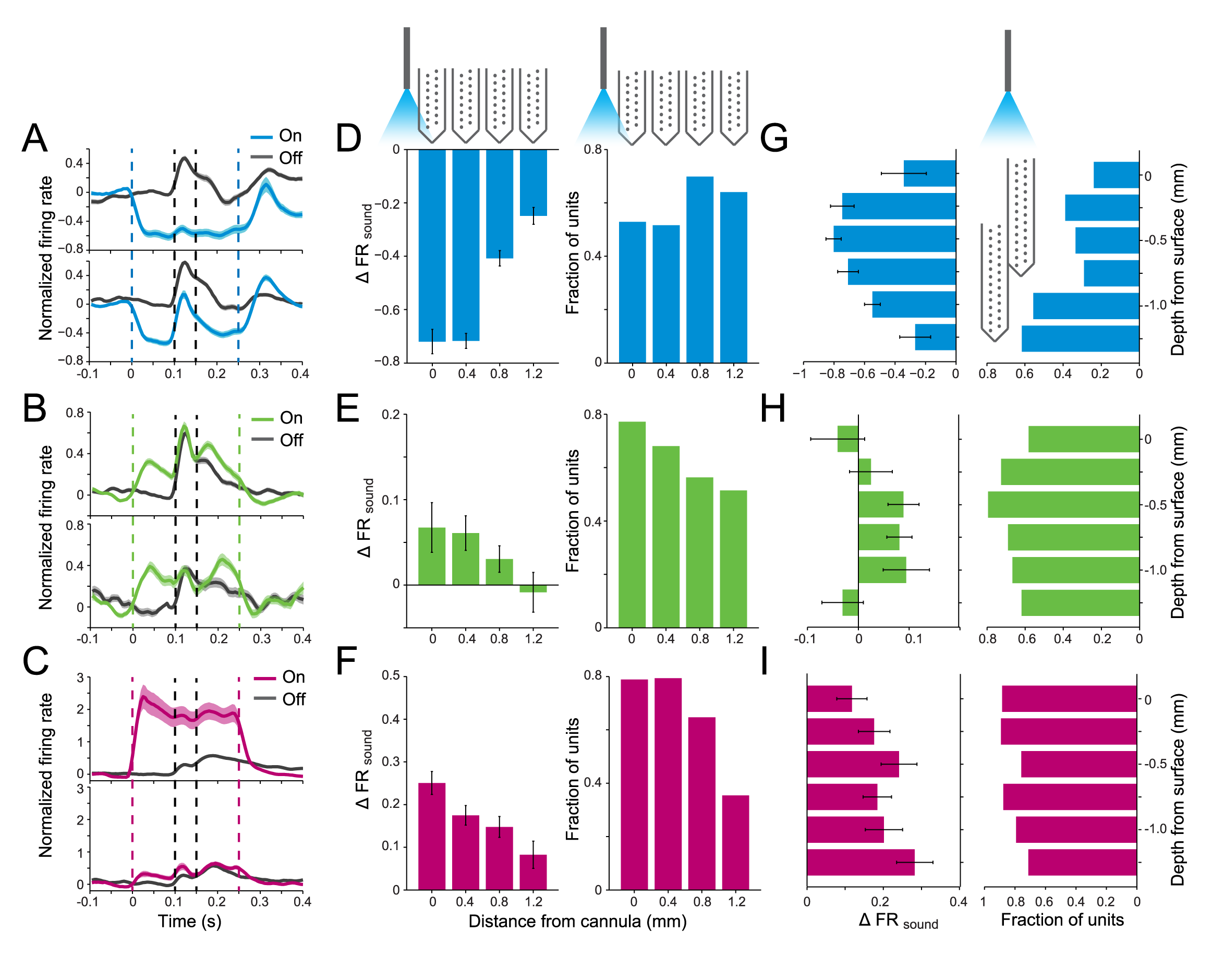

Supplement: S2 Fig — We measured the effect of light activation on multiunit activity during noise bursts. We recorded neuronal activity in the AC of head-fixed awake mice at 0, 0.4, 0.8 and 1.2 mm from the optocannula and at 6 depths between 0 and 1.25 mm from brain surface. The sound stimulus was a 50 ms long white noise burst. On half the trials, the sound was accompanied by a 250 ms long light pulse emitted from the optocannula, which started 100 ms prior to sound onset. Multiunit clusters were identified using Plexon online spike-sorter, and their firing rate was computed on light-off and light-on trials (S2A-SC Fig). We computed the percentage of units whose baseline firing rate (0–50 ms pre light onset) was increased (PV-Arch and CamKIIα-ChR2 groups) or decreased (PV-ChR2) due to light (0–50 ms post light onset) and the index of change of their mean firing rate during white-noise burst (0–50 ms post noise burst onset) on light-on as compared to on light-off trials (ΔFRsound). In all three groups, the effect of light on sound-evoked multiunit activity significantly declined over distance (S2D-S2F Fig one-way ANOVA with distance as factor, PV-ChR2: F 3,535 = 52.28, p = 1.3e-29; PV-Arch: F 3,437 = 3.34, p = 0.019; CamKIIα-ChR2: F 3,555 = 4.26, p = 0.005). The effect of light was stronger in the CamKIIα-ChR2 than in PV-Arch group across all distances (two-way ANOVA: effect of group, F(df = 1) = 34.24, p < 0.0001; effect of distance, F(df = 3) = 4.58, p = 0.0038; interactions, group x distance, p > 0.05). The effect of the light on multiunit activity as a function of depth was heterogeneous (S2D-S2F Fig). In all three groups, one-way ANOVA with depth as factor was not significant. In PV-ChR2 and PV-Arch groups, change in FR exhibited an inverted U-shape dependency on the depth,declining significantly from 0.5–0.75 mm to 1.25 mm (two-sample t test, PV-ChR2: t 101 = −5.3, p = 8.3e-7; PV-Arch: t 190 = 2.1, p = 0.039). For CamKIIα-ChR2 mice, the dependency exhibited a more linear pattern [file pbio.1002308.s003.tif]

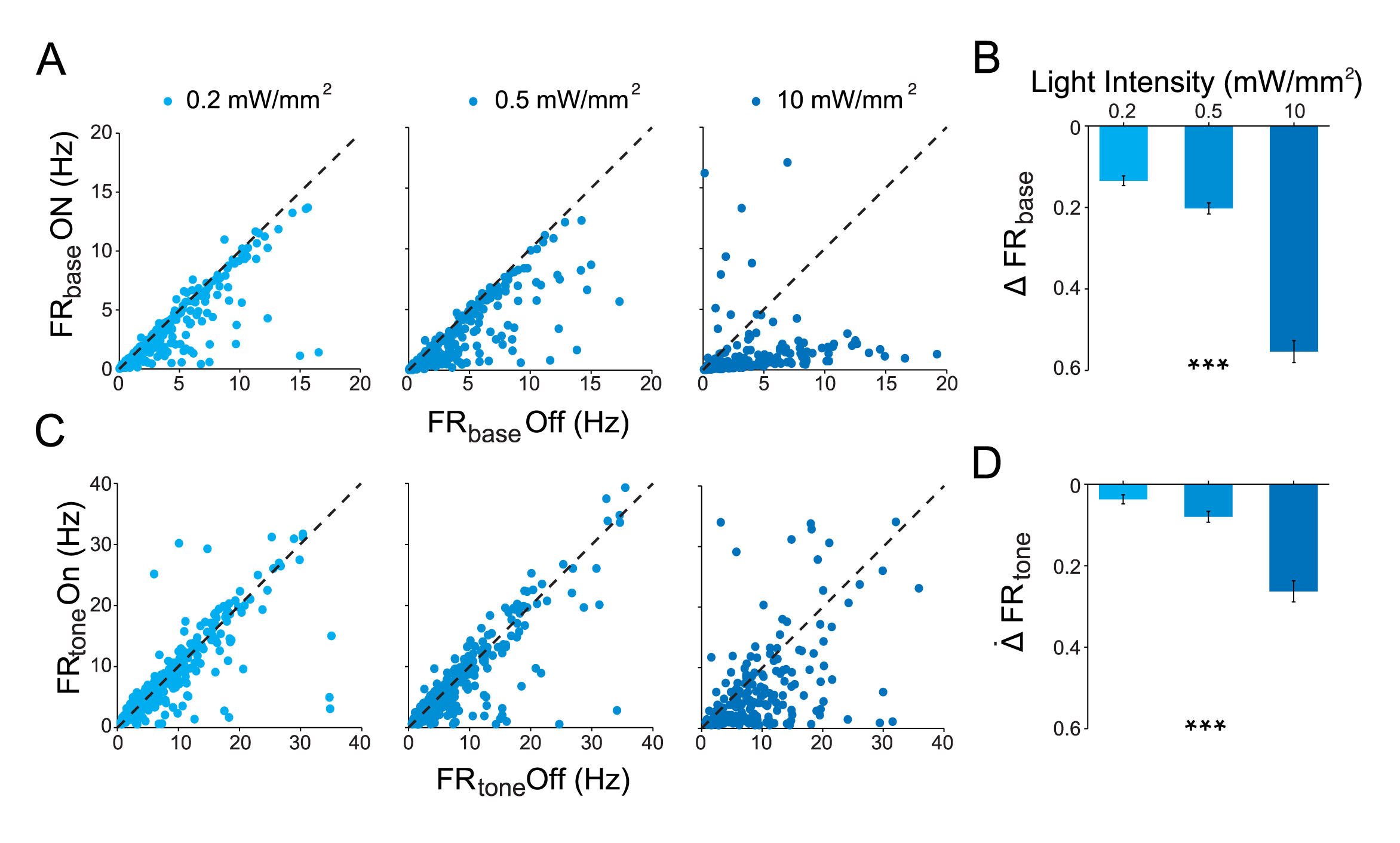

Supplement: S3 Fig — (A) Light intensity. Left: 0.2 mW/mm2 (n = 330 neurons); middle: 0.5 mW/mm2 (n = 322 neurons); right: 10 mW/mm2 (n = 202 neurons). Each circle represents a single unit. Spontaneous firing rate (FRbase) was suppressed as result of photoactivation of PVs in a light-intensity-dependent fashion (FRbase on light-On is plotted versus light-Off trials). (B) Mean index of change in spontaneous firing rate due to different intensity of photoactivation of PVs over neuronal population. ***: One-way ANOVA, F 2,851 = 156.38, p = 1.5e-58. (C) Tone-evoked firing rate (FRtone) is suppressed during photostimulation (light-On versus light-Off trials). Columns as in (A). (D) Mean index of change in FRtone due to different intensity of photostimulation of PVs over neuronal population. ***: One-way ANOVA, F 2,851 = 48.35, p = 1.3e-20. (TIF) [file pbio.1002308.s004.tif]

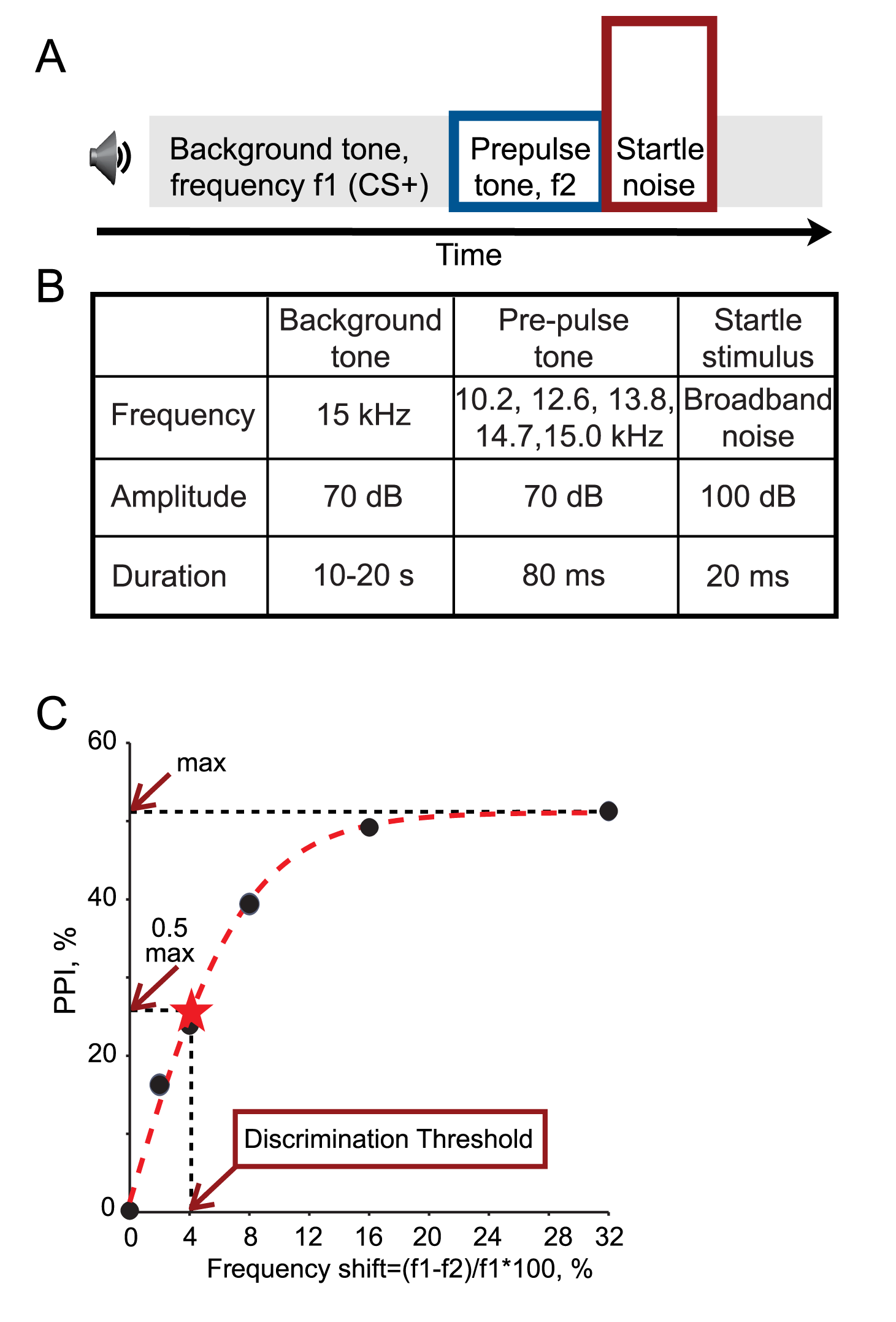

Supplement: S4 Fig — The test relied on measurement of inhibition of auditory startle response by PPI. (A) Time course of acoustic stimulation during a single PPI trial. Three stimuli were presented in succession: 1) background tone at frequency (f1) identical to CS+ used in fear conditioning; 2) prepulse tone at the same amplitude but different frequency (f2) than the background tone; 3) startle broadband noise that evoked a startle response. (B) Parameters of stimuli used in PPI. Note that the duration of the background tone varied randomly between 10 and 20 s. On each trial, prepulse tone was presented at a frequency randomly selected from five listed frequencies. (C) Sample PPI versus Tone frequency shift curve. Reduction in the magnitude of the startle response (% PPI) increased as a function of frequency shift (%) between the background and prepulse tone. Each data point represents the average PPI over at least ten trials. Red dashed line is the logistic fit curve (see Methods). Th was defined as the frequency shift at 50% maximum PPI. (TIF) [file pbio.1002308.s005.tif]

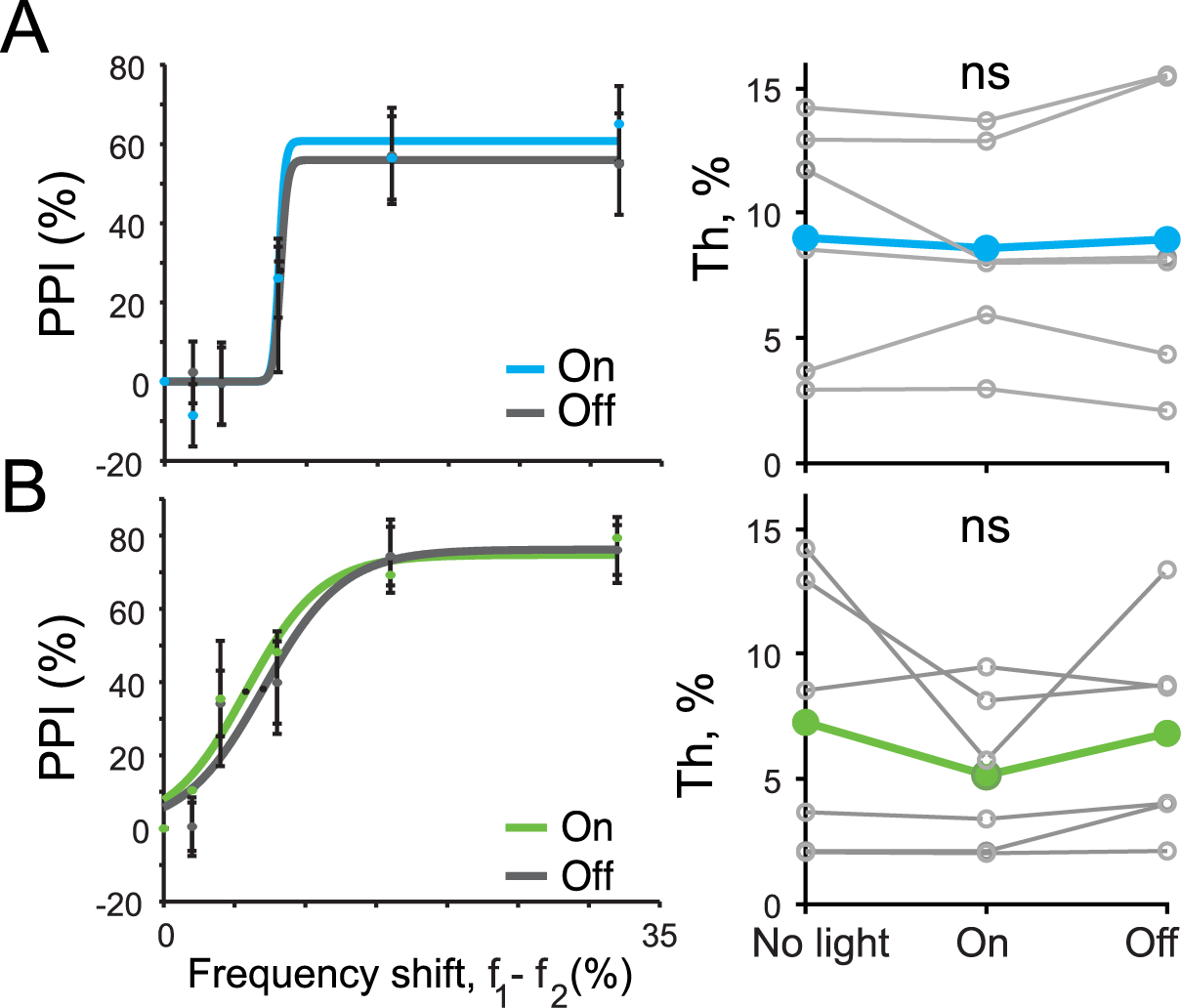

Supplement: S5 Fig — (A) Behavioral frequency discrimination acuity as measure by Th is not affected by photostimulation with blue light in mice expressing control viral constructs (n = 6). Left. PPI as a function of frequency shift in light-On (blue) and light-Off (black) condition. Right. Mean Th values (blue) and Th for each subject (gray) in light-On and light-Off condition, and in the session where no photostimulation was presented (“No light”). (B) Behavioral Th is not affected by photostimulation with green light in mice expressing control viral constructs (n = 6). Left. PPI as a function of frequency shift in light-On (green) and light-Off (black) condition. Right. Mean Th values (green) and Th for each subject (gray) in light-On and light-Off condition, and in the session where no photosuppression was presented (“No light”). Axes: same as in Fig 2C and 2E. Blue light: paired t test, t5 = −0.55, p = 0.1. Green light: paired t test, t 5 = −1.35, p = 0.24. (TIF) [file pbio.1002308.s006.tif]

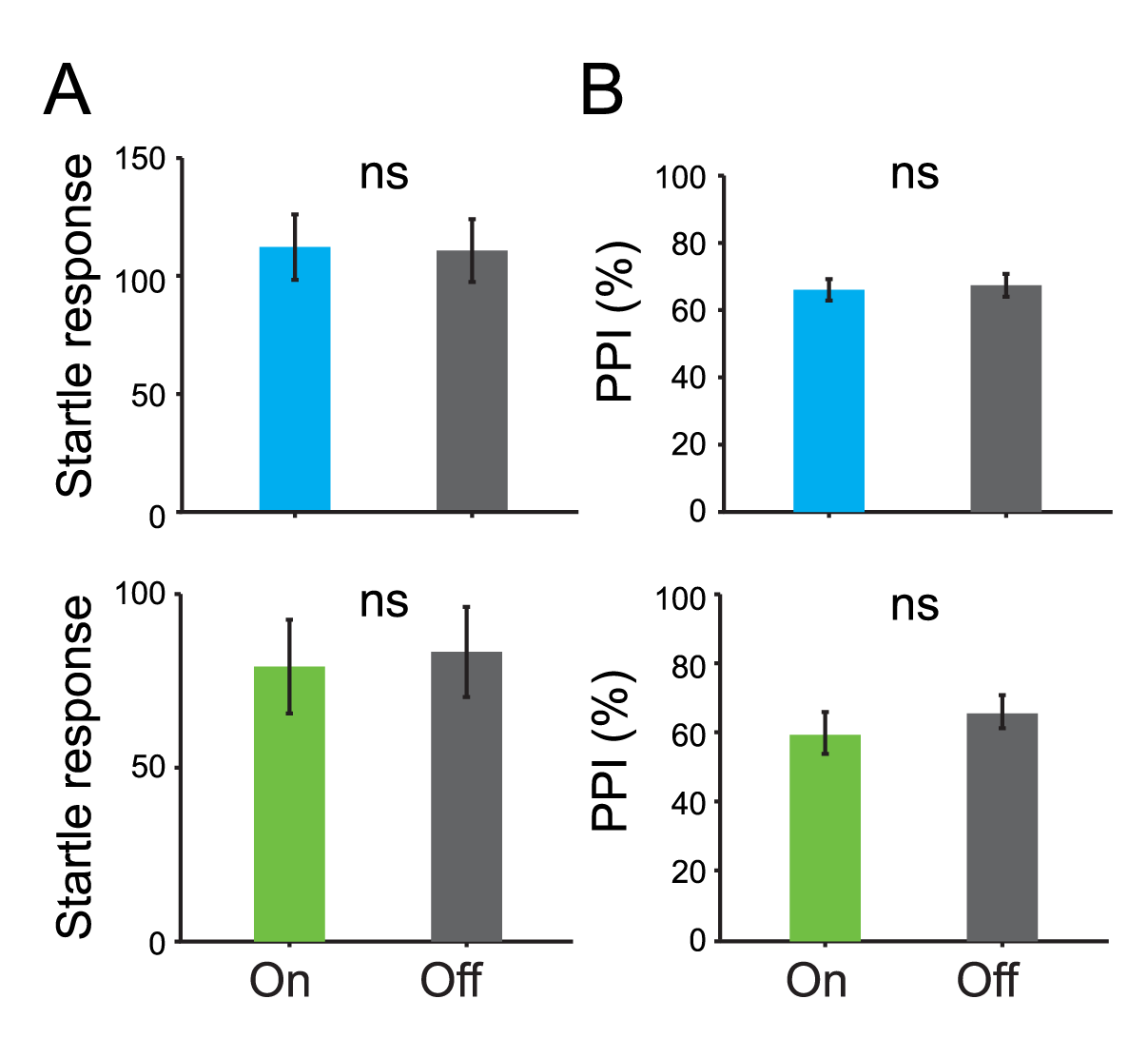

Supplement: S6 Fig — (A) Startle response magnitude in the absence of prepulse signal (no frequency shift between background and prepulse tones) on light-On (color bars) and light-Off trials (gray bars) averaged across mice from PV-ChR2 and PV-Arch groups. ns: Difference not significant (paired t test. PV-ChR2: n = 20, t 19 = 0.365, p = 0.719; PV-Arch: n = 16 t 15 = −0.86, p = 0.41). (B) Maximum PPI values induced by prepulse frequency shift on light-On (color bars) and light-Off trials (gray bars) averaged across mice from PV-ChR2 and PV-Arch groups. ns: Difference not significant (PV-ChR2: paired t test, t 19 = −0.63, p = 0.535; PV-Arch: paired t test, t 15 = −1.9, p = 0.083). Each bar represents average across subjects ± SEM. (TIF) [file pbio.1002308.s007.tif]

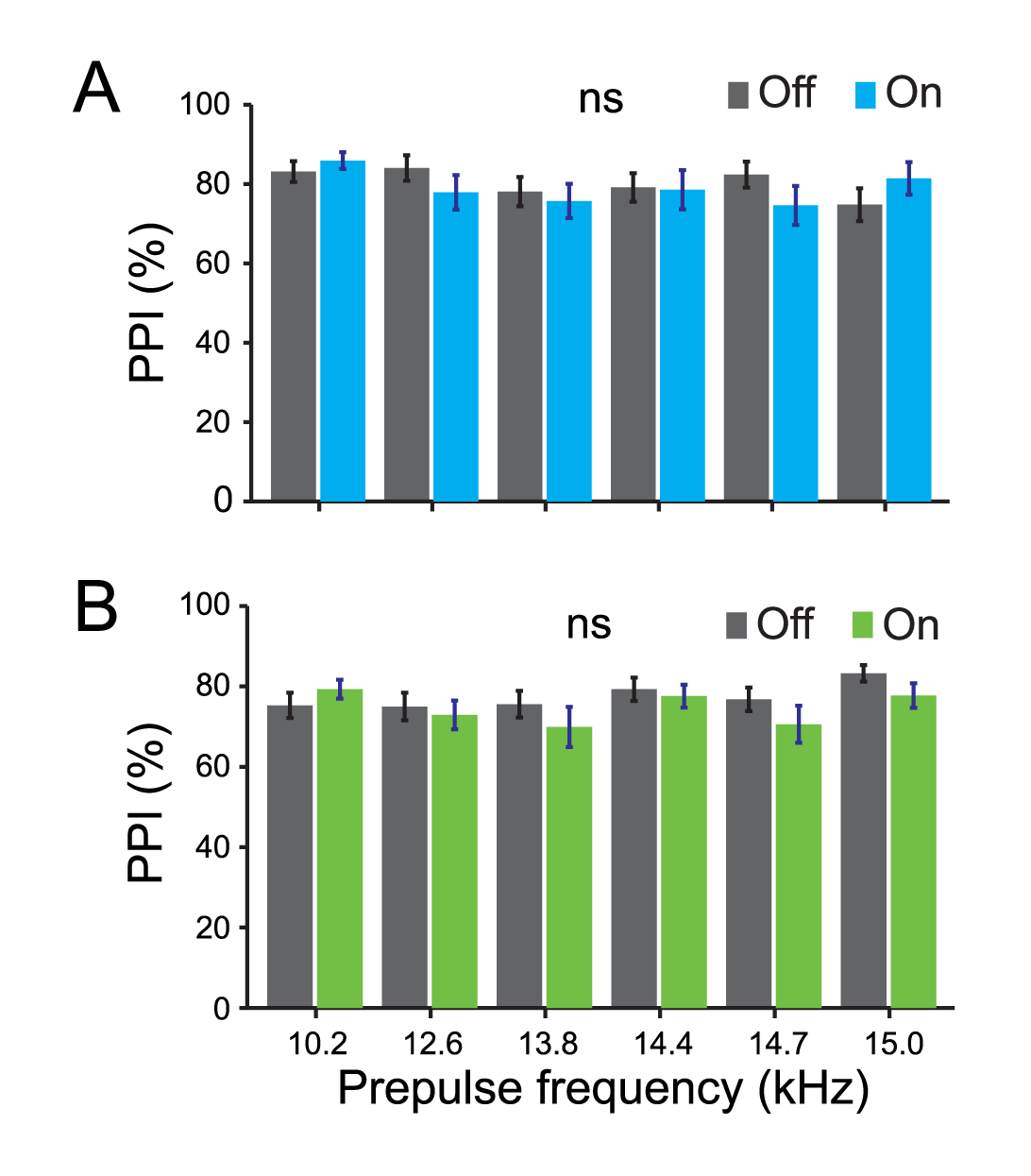

Supplement: S7 Fig — Neither activation (A) nor inhibition (B) of PV interneurons affected PPI induced by tones of different frequency without background tone. These results indicate that photostimulation did not change subjective loudness of tones in frequencies used in frequency discrimination test. Comparison of PPI for light-On (color bars) and light-Off trials (gray bars) revealed no significant difference in either group (two-way ANOVA-light effect, PV-ChR2: n = 598 trials, F 1,586 = 0.52, p = 0.47; PV-Arch: n = 602 trials, F 1,590 = 2.1, p = 0.14). Comparison of PPI elicited by prepulse tones of six different frequencies did not reveal significant difference (two-way ANOVA-frequency effect, PV-ChR2: F 5,586 = 0.90, p = 0.47; PV-Arch: F 5,590 = 1.7, p = 0.14). (A) Data for mice from PV-ChR2 group (n = 4 mice). (B) Data for mice from PV-Arch group (n = 5 mice). Each bar represents mean ± SEM across subjects. (TIF) [file pbio.1002308.s008.tif]

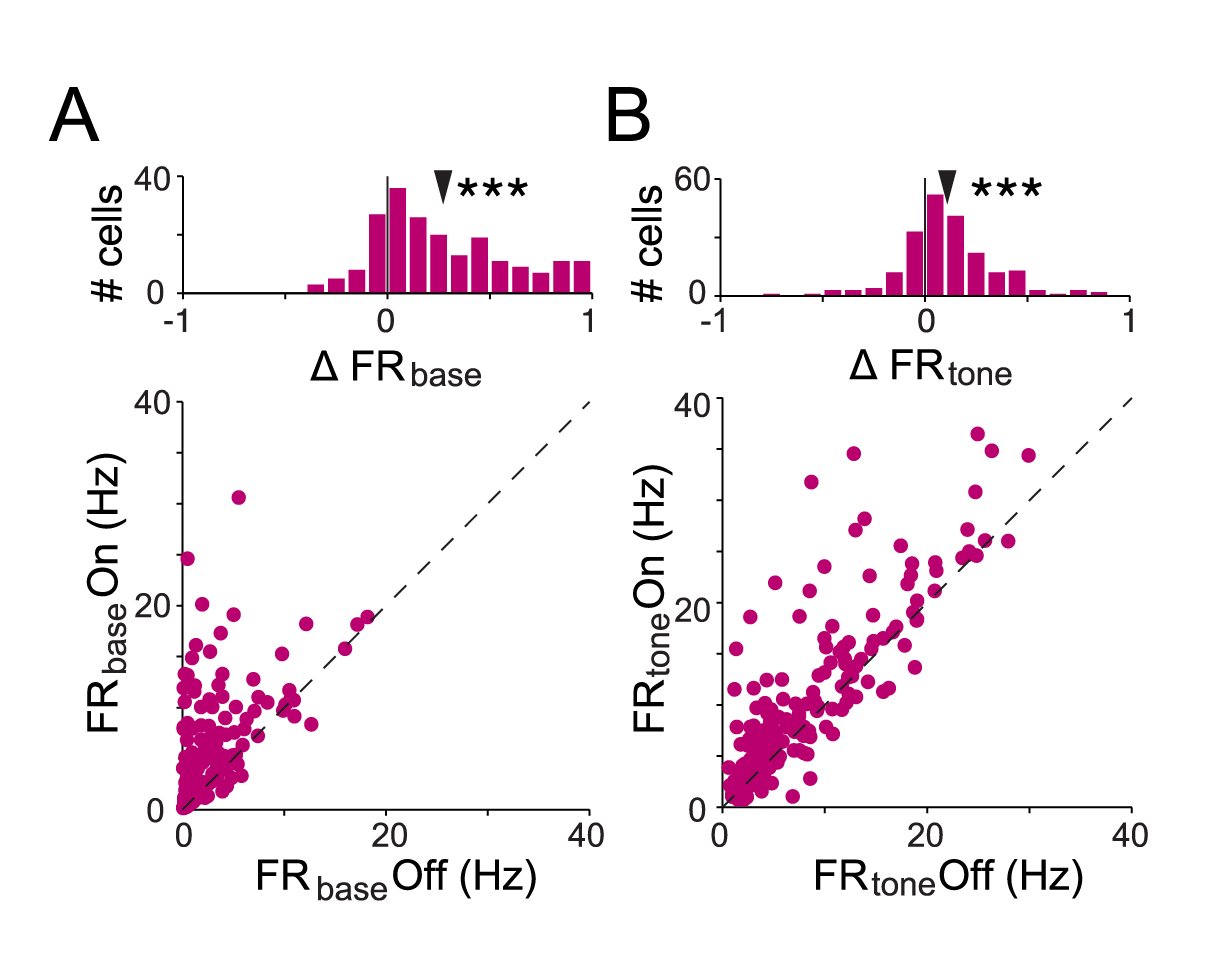

Supplement: S8 Fig — (A) Photoactivation of CamKIIα neurons leads to a significant increase in FRbase of putative excitatory neurons. Top: index of change in the FRbase across neuronal population. Bottom: FRbase in light-On trials versus light-Off trials. ***: one-sample t test, t 205 = 11.84, p = 5.4e-25, mean ΔFRbase = 0.27. (B) Photostimulation increased the tone-evoked firing rate. Top: histogram of the index of change in the tone-evoked firing rate (FRtone) across neuronal population. Bottom: FRtone in light-On trials plotted versus light-Off trials. ***: t 205 = 6.71, p = 1.9e-10, mean ΔFRtone = 0.11. (TIF) [file pbio.1002308.s009.tif]

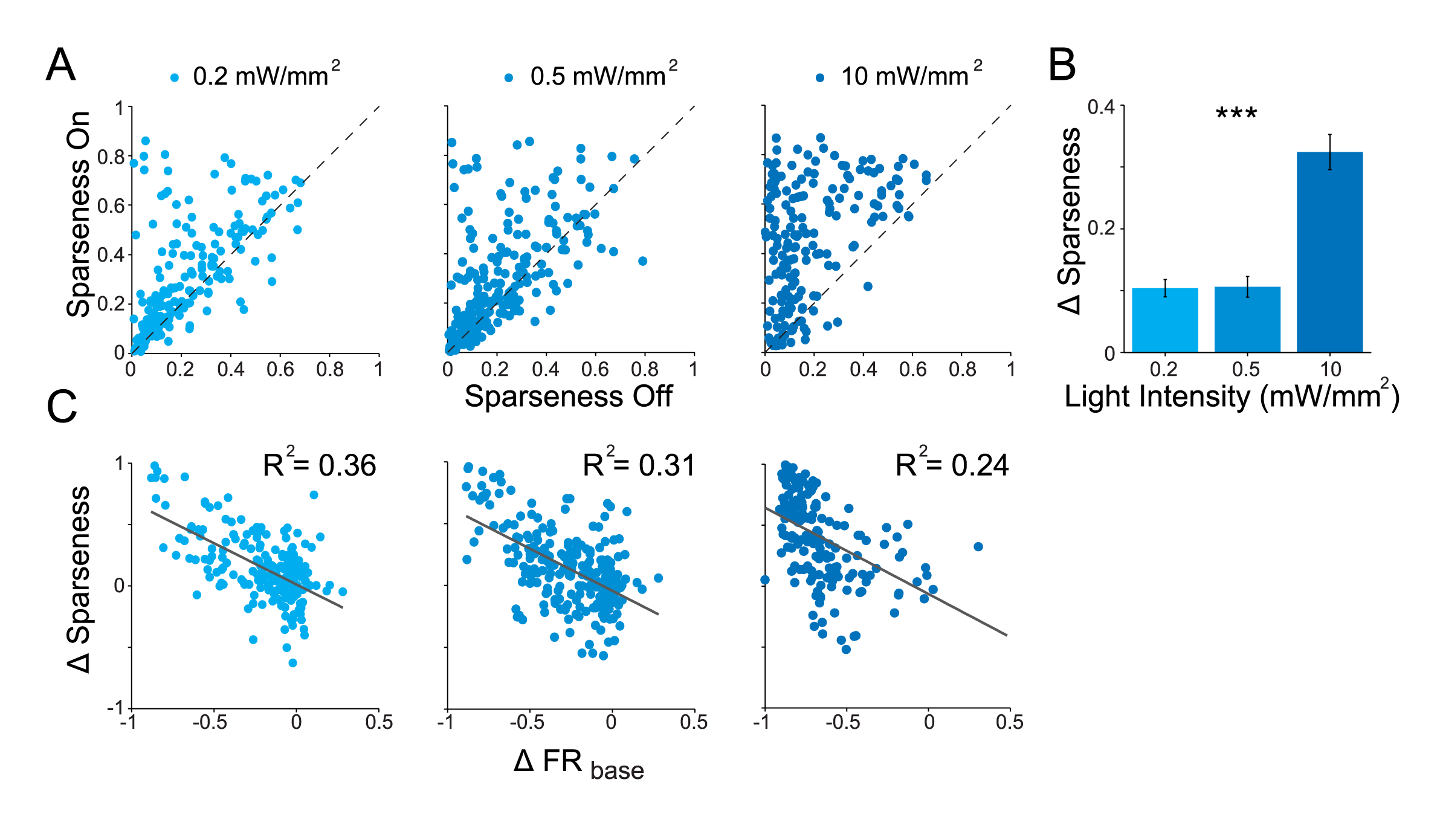

Supplement: S9 Fig — (A) Light intensity. Left: 0.2 mW/mm2 (n = 215 neurons); middle: 0.5 mW/mm2 (n = 240 neurons); right: 10 mW/mm2 (n = 175 neurons). Each circle represents a single auditory unit suppressed by light. Sparseness of tuning increased due to photoactivation of PVs in a light intensity-dependent fashion (sparseness on light-On is plotted versus light-Off trials). (B) Mean index of change in sparseness during photostimulation of PVs at varying light intensities. ***: One-way ANOVA, F2,851 = 38.2, p = 1.3e-16. (C) Index of change in sparseness of neuronal responses resulting from different levels of laser stimulation as a function of change in FRbase. 0.2 mW/mm2: p = 1.5e-23; 0.5 mW/mm2: p = 4.2e-21; 10 mW/mm2: p = 8.9e-12. Columns as in (A). (TIF) [file pbio.1002308.s010.tif]

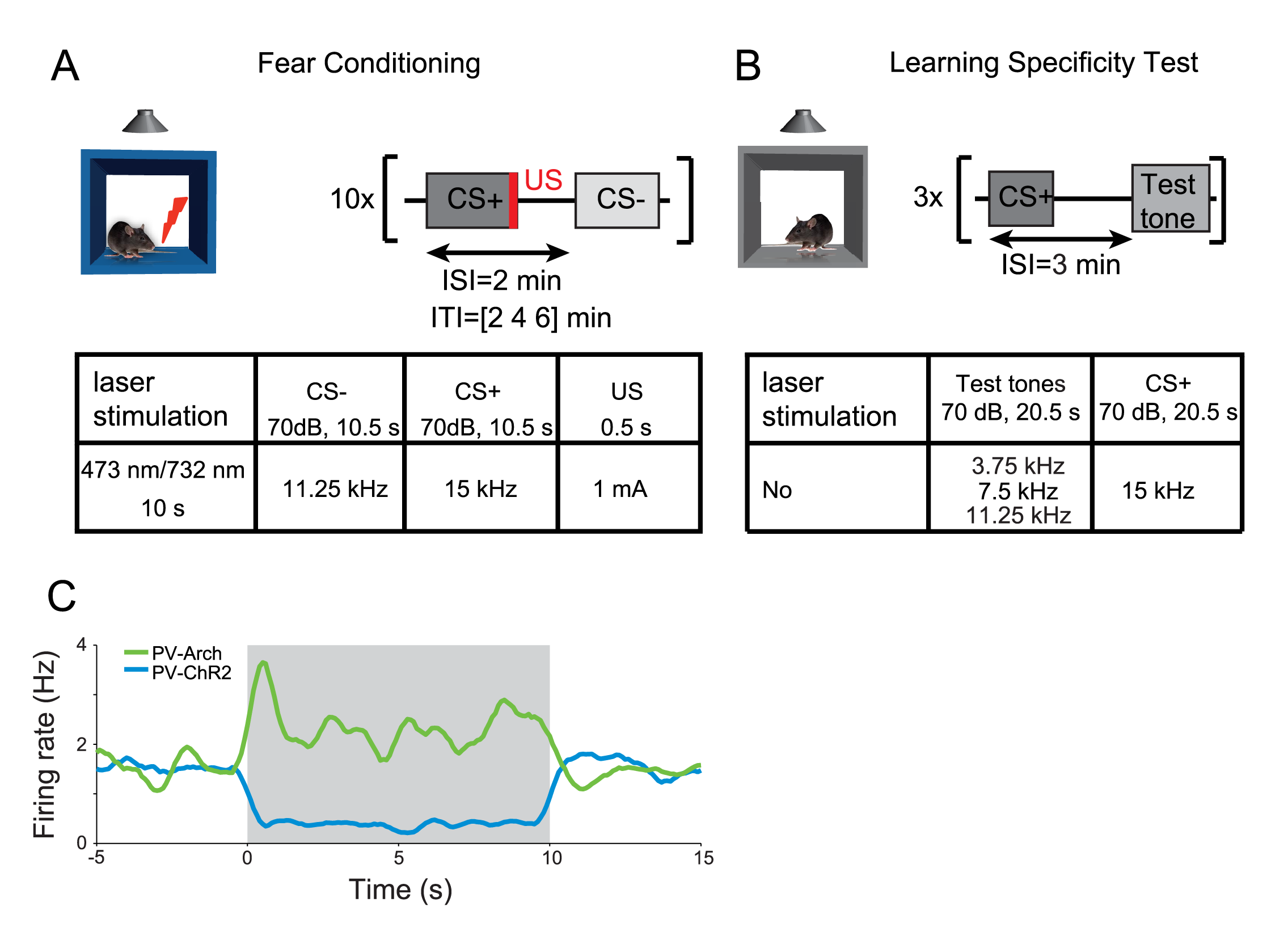

Supplement: S10 Fig — (A) A discriminative fear conditioning session consisted of 10 presentations of a 15 kHz tone (CS+) coterminated with a mild foot-shock (unconditioned stimulus, US). In addition, 10 unpaired tones (11.25 kHz, CS) were presented along with CS+ with 2 min interstimulus interval (ISI). Randomized inter-trial interval (ITI, time between CS+ presentations) was 2,4, or 6 min. (B) The LS test was carried out in a different context from conditioning. The LS test consisted of CS+ and three test tones (3.75, 7.5, 11.25 kHz), presented in random order three times each at 3 min ISI. LS was assayed as the differential freezing response to CS+ and test tones (Methods). (C) Peristimulus time histogram (PSTH) of putative PV- neurons in response to 10-s-long laser pulses (outlined by gray rectangle) in PV-Arch (green) and PV-ChR2 (blue) mice. (TIF) [file pbio.1002308.s011.tif]

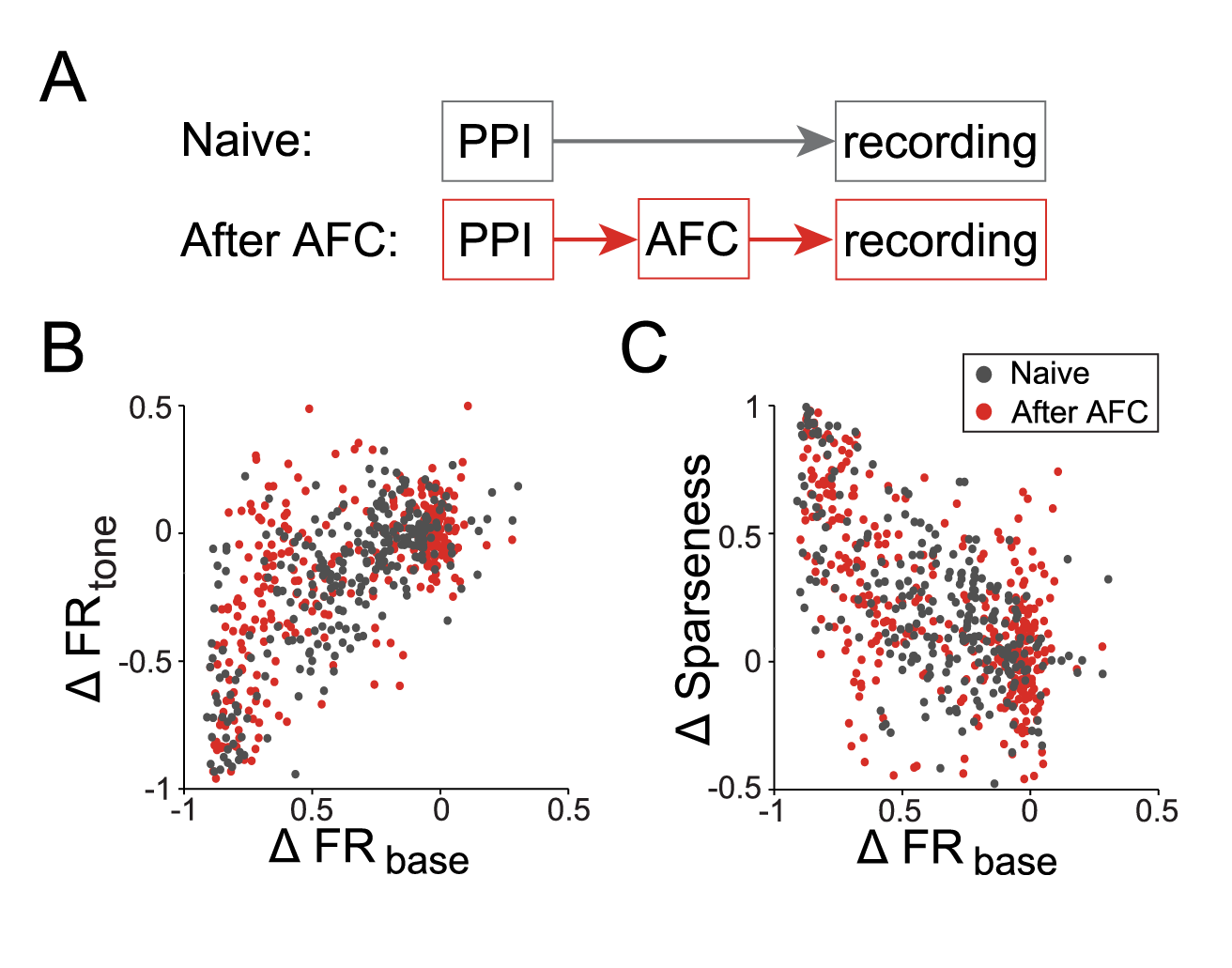

Supplement: S11 Fig — (A) Diagram of experimental procedure for naïve animals (top) and animals that underwent DAFC prior to recording. Change in spontaneous and tone-evoked firing rate (B) and change in sparseness of neuronal tuning due to photoactivation of PVs (C) were similar for mice that underwent DAFC and naïve animals (MANOVA with conditioned or naive subject as a factor (F1,628 = 1.0, p = 0.49)). Gray dots: results of electrophysiological recording from each neuron in naive subjects (n = 6). Red dots: results from electrophysiological recording from each neuron 2–5 days after fear conditioning (n = 8). (TIF) [file pbio.1002308.s012.tif]

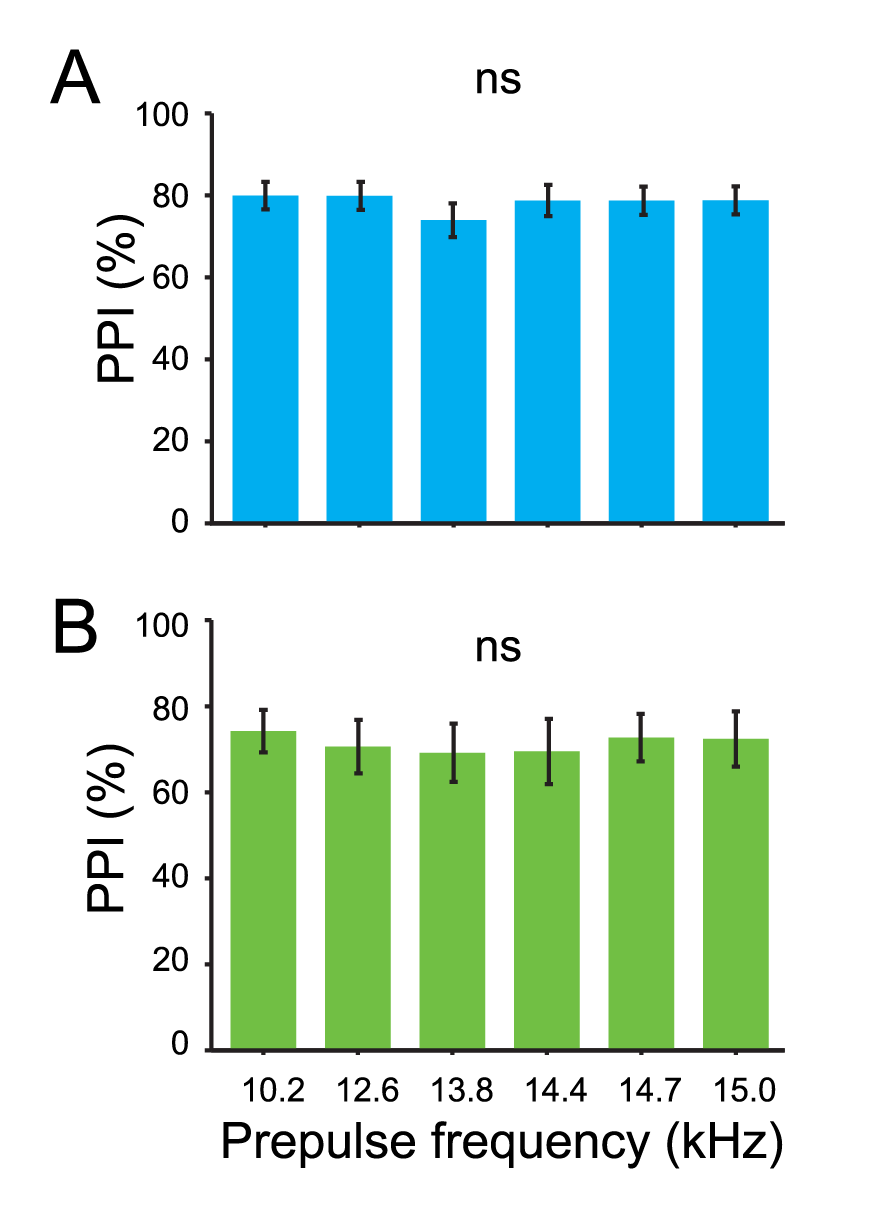

Supplement: S12 Fig — Perception of tone loudness was estimated as PPI elicited by prepulse tone without background tone. Each bar represents mean ± SEM across mice in PV-ChR2 group (A, n = 20, repeated-measures ANOVA, p = 0.066) and PV-Arch group (B, n = 16, p = 0.52). (TIF) [file pbio.1002308.s013.tif]

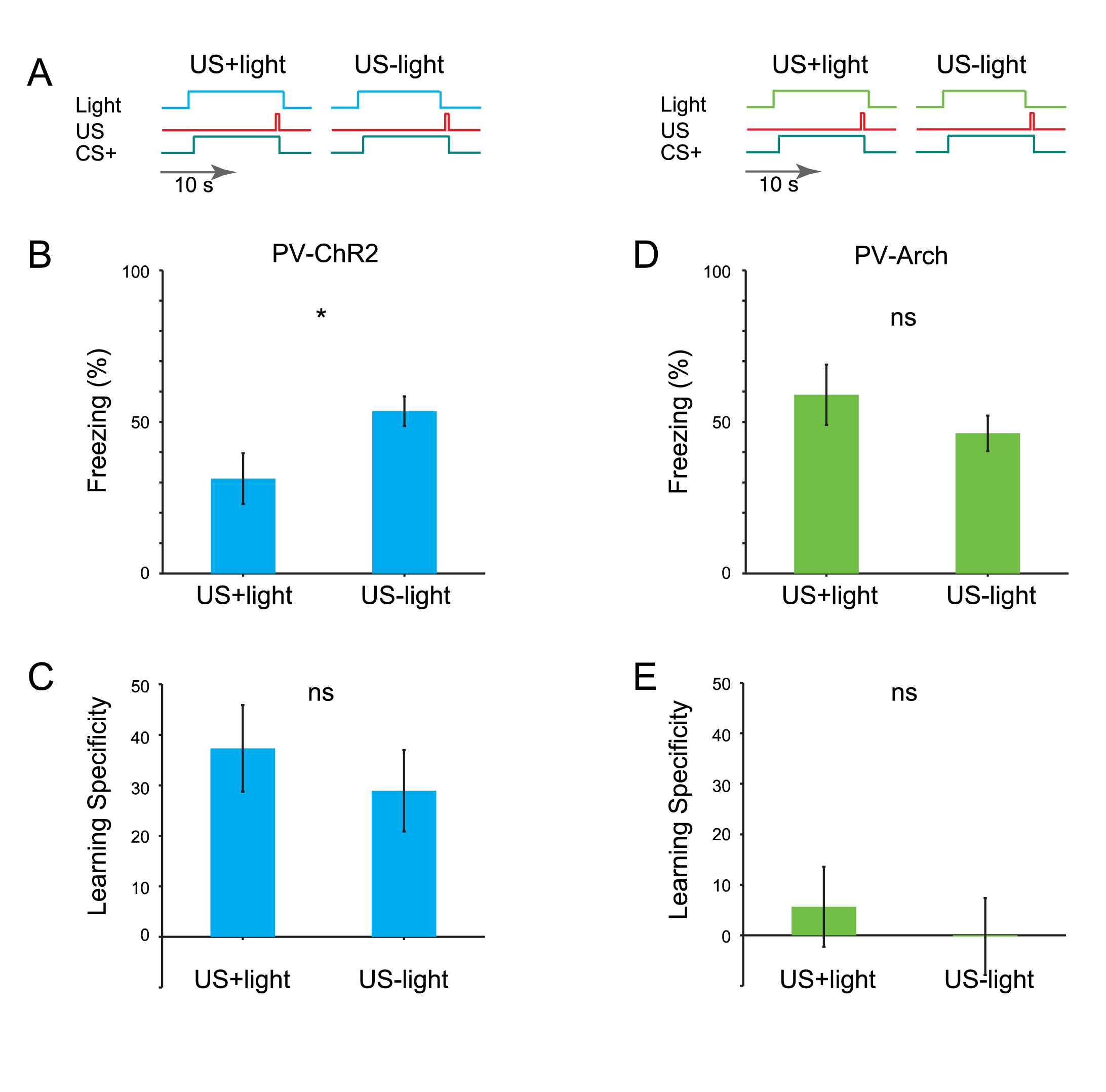

Supplement: S13 Fig — (A) In US+light group, blue (left) or green (right) light stimulation was 11 s long and overlapped with the presentation of electric foot-shock (US). In US-light group, light stimulation lasted for 10 s and terminated 0.5 s before the US onset. (B) In PV-ChR2 group, activation of PVs during US presentation (US+light) significantly reduced freezing during test session as compared to US-light group. *: p = 0.025, t test, t 14 = 2.53,. (C) In PV-ChR2 group, activation of PVs during US presentation (US+light) did not affect specificity of conditioned response. ns: t test, t 14 = 0.67, p = 0.51. (D and E) In PV-Arch group, inhibition of PVs during US presentation (US+light) did not significantly affect either freezing (d) (t test, t 11 = 1.16, p = 0.27) or LS (e) (t test, t 11 = 0.45, p = 0.66). (TIF) [file pbio.1002308.s014.tif]

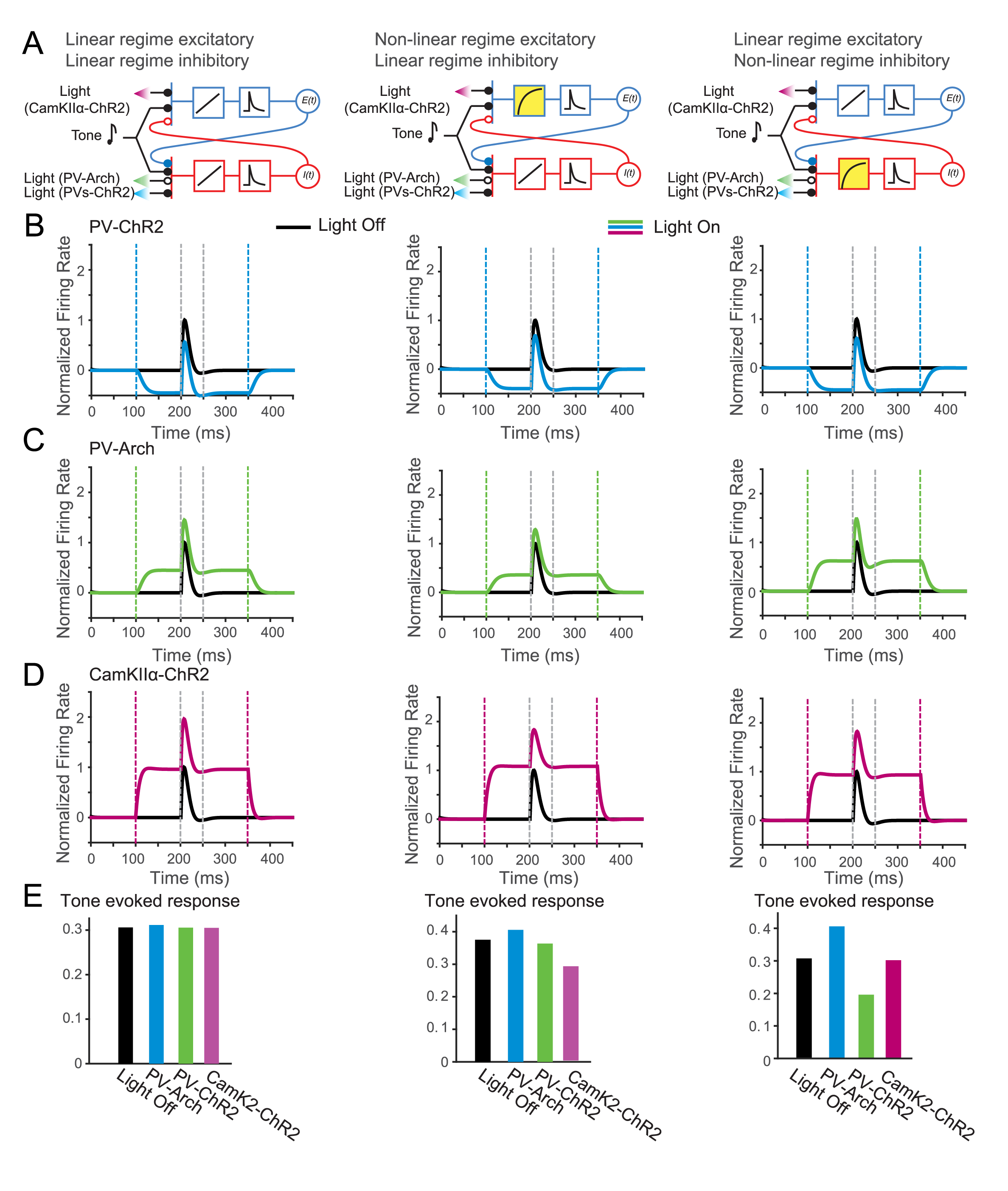

Supplement: S14 Fig — (A) Three additional models that were used to model the excitatory-inhibitory coupled networks. Left: the synaptic transfer function was modeled as linear for both excitatory and inhibitory population. Center: The synaptic transfer function was modeled as saturating for excitatory, and linear for inhibitory population. Right: The synaptic transfer function was modeled as saturating for inhibitory and linear for excitatory population. (B–D) Responses of excitatory neuronal population to a 50-ms long tone presented at 200 ms with (color) and without (black) optogenetic stimulation (color, btw. 100–350 ms). B. Blue: activation of inhibitory neurons. C. Green: suppression of inhibitory neurons. D. Magenta: Activation of excitatory neurons. Compare to Fig 3A, 3C and 3E. (E) Mean tone-evoked magnitude (mean firing rate during tone—spontaneous firing rate just preceding the tone). Colors as in B–D. Compare to Fig 3B, 3D and 3F. (TIF) [file pbio.1002308.s015.tif]
